# A new electric method for non-invasive continuous monitoring of stroke volume and ventricular volume-time curves

**DOI:** 10.1186/1475-925X-11-51

**Published:** 2012-08-17

**Authors:** Maurits K Konings, Henk G Goovaerts, Maarten R Roosendaal, Rienk Rienks, Ferry M Koevoets, Ronald L Bleys, Wolfgang F Buhre, Paul M Dorresteijn, Tim Hesselink, Arthur E Officier, Charles L Hollenkamp, Frank E Rademakers

**Affiliations:** 1 Dept. of Medical Technology, University Medical Center Utrecht, Utrecht, The Netherlands

## Abstract

**Background:**

In this paper a new non-invasive, operator-free, continuous ventricular stroke volume monitoring device (Hemodynamic Cardiac Profiler, HCP) is presented, that measures the average stroke volume (SV) for each period of 20 seconds, as well as ventricular volume-time curves for each cardiac cycle, using a new electric method (Ventricular Field Recognition) with six independent electrode pairs distributed over the frontal thoracic skin. In contrast to existing non-invasive electric methods, our method does not use the algorithms of impedance or bioreactance cardiography. Instead, our method is based on specific 2D spatial patterns on the thoracic skin, representing the distribution, over the thorax, of changes in the applied current field caused by cardiac volume changes during the cardiac cycle. Since total heart volume variation during the cardiac cycle is a poor indicator for ventricular stroke volume, our HCP separates atrial filling effects from ventricular filling effects, and retrieves the volume changes of only the ventricles.

**Methods:**

ex-vivo experiments on a post-mortem human heart have been performed to measure the effects of increasing the blood volume inside the ventricles in isolation, leaving the atrial volume invariant (which can not be done in-vivo). These effects have been measured as a specific 2D pattern of voltage changes on the thoracic skin. Furthermore, a working prototype of the HCP has been developed that uses these ex-vivo results in an algorithm to decompose voltage changes, that were measured in-vivo by the HCP on the thoracic skin of a human volunteer, into an atrial component and a ventricular component, in almost real-time (with a delay of maximally 39 seconds). The HCP prototype has been tested in-vivo on 7 human volunteers, using G-suit inflation and deflation to provoke stroke volume changes, and LVot Doppler as a reference technique.

**Results:**

The ex-vivo measurements showed that ventricular filling caused a pattern over the thorax quite distinct from that of atrial filling. The in-vivo tests of the HCP with LVot Doppler resulted in a Pearson’s correlation of R = 0.892, and Bland-Altman plotting of SV yielded a mean bias of -1.6 ml and 2SD =14.8 ml.

**Conclusions:**

The results indicate that the HCP was able to track the changes in ventricular stroke volume reliably. Furthermore, the HCP produced ventricular volume-time curves that were consistent with the literature, and may be a diagnostic tool as well.

## Background

In intensive care medicine, the measurement of left ventricular stroke volume (SV) and cardiac output (CO) is important in the hemodynamic management of peri-operative and critically ill patients. Pulmonary artery thermodilution CO monitoring using the pulmonary artery catheter has major disadvantages, because pulmonary artery catheterization is time-consuming, and associated with a risk of morbidity and mortality [1]. Noninvasive ultrasound Doppler techniques to determine CO have been developed, but require skilled operators, and therefore are not suited for monitoring CO continuously. More recently, esophageal Doppler, Fick principle applied to carbon dioxide, and pulse contour analysis have been identified as key technologies for cardiac output monitoring [2,3]. Furthermore, recent advances in MRI show promising results in measuring cardiac output [4,5]; MRI however is not suited for continuous bed-side monitoring. Stroke volume can also be measured reliably using the conductance catheter [6]. This method however requires a catheter to be placed in the left ventricle. The transcardiac conductance method produces satisfactory results in a less invasive manner, but still requires an internal central venous electrode [7]. Thoracic impedance cardiography (ICG) is a non-invasive technique, but conflicting results concerning the validity and reliability of ICG have been reported, varying from satisfactory correlations [8-10] to poor correlations [11,12] in comparison to thermodilution CO measurements. Bioreactance cardiography [13] provides better results than ICG, because it measures also phase shifts instead of merely amplitudes of AC voltages, and bioreactance cardiography is currently being evaluated in clinical settings. All in all however, there is still a need for a non-invasive, precise, continuous, and operator-free method of CO measurement.

Recently, such a method (which we will refer to as ’Ventricular Field Recognition’, VFR) has been developed in our group [14]. In a previous paper [15], we presented an animal study, in which the cardiac output readings using the new VFR method were compared to the cardiac output readings from a flow probe around the aorta, resulting in a bias of - 0.114 L/minute and a variability of the bias (2 standard deviations, 2SD) of 0.55 L/minute in a Bland-Altman analysis; without however providing any mathematical details about the principle of operation of the new method. In the present paper, the physics and mathematics of the principle of operation of the VFR method is presented, and some basic assumptions underlying the VFR method are analysed and investigated. Furthermore, we present the results of a validation study of the VFR method on *human* volunteers.

The Ventricular Field Recognition method is based on our findings that, (i) if a weak, harmless, constant AC electric current is applied over the thorax, the emptying and filling of the *ventricles* during the cardiac cycle gives rise to a specific two-dimensional spatial pattern of changes in current density over the frontal thoracic skin during the cardiac cycle, and, furthermore, (ii) that this pattern is distinctly different from the spatial pattern of current density changes due to the filling and emptying of the *atria*. This is caused by the fact that the ventricles are situated at other locations inside the thorax than the atria, and hence, the location (on the frontal thoracic skin) of the “ventricular epicenter” of the changes in the applied current field, due to ventricular volume changes, differs from the location of the “atrial epicenter” corresponding to atrial volume changes.

The changes, during a cardiac cycle, in current density distribution at the surface of the thorax can be measured as voltage changes using multiple skin electrodes, distributed over the thoracic skin. Evidently, simultaneously with the ventricular volume changes during the cardiac cycle, some atrial volume changes take place as well. Therefore, when measuring in-vivo on a patient or volunteer, the measured 2D pattern of voltage changes distributed over the skin of the thorax, is a superposition of three different patterns, viz. (i) the pattern due to volume changes in the atria, (ii) the pattern due to volume changes in the ventricles, and (iii) a pattern due to extracardial effects, such as e.g. diameter changes in large blood vessels outside the heart. In order to monitor the stroke volume and ventricular volume-time curves of a patient however, the volume changes in exclusively the ventricles have to be established (because total heart volume variation is a poor indicator of ventricular stroke volume [16]). Therefore, the Ventricular Field Recognition method presented in this paper has been designed to distill the ventricular volume changes from the measured total superposition of the three patterns. In order to do so, the specific pattern of voltage changes on the frontal thoracic skin associated with volume change in *only* the ventricles, needs to be known.

Therefore, an extensive set of *ex-vivo* measurements has been performed in our group, using *post-mortem* human hearts and thoraces. The valves inside the post-mortem human hearts were sealed, and each compartment of the heart was connected to a silicon tube leading to a large syringe, thus enabling the separate filling (or emptying) of e.g. *only *the ventricles, or *only* the atria, as will be explained in more detail in section Methods: ex-vivo post-mortem calibration. The effect of the filling action on the applied thoracic current density distribution was registered by a matrix of electrodes on the skin of the post-mortem thorax. In this way, a distinct 2D pattern on the frontal thoracic skin was measured, representing the voltage changes due to a specific standardized filling action of only the ventricles with a fixed known volume. We will refer to this specific spatial 2D pattern as the “ventricular fingerprint”. Furthermore, we measured an “atrial fingerprint” as well, representing a standardized filling of only the atria. Throughout this paper, the word “fingerprint” refers to the measurements performed *ex-vivo* on human *post-mortem* cadaveric materials.

In the *in-vivo* situation, with a beating heart inside the thorax of a patient, the actual voltage change pattern that is measured over the frontal thoracic skin, is a linear combination of these *post-mortem* ventricular and atrial fingerprints, because volume changes take place in ventricles *and* atria simultaneously during a cardiac cycle in-vivo. Let *τ* denote the time (in milliseconds), during one single cardiac cycle, that has lapsed after the last R-peak of the ECG. The above mentioned linear combination may then be written as:

(1) $$\begin{array}{lll} \;\left[\text{in-vivo voltage}(x,\tau )\right] & -\left[\text{in-vivo voltage}(x,0)\right]\;=\alpha (\tau )\left[\text{atrial postmortem fingerprint}(x)\;\right]\; & \;\\ & +\psi (\tau )\left[\text{ventricular postmortem fingerprint}(x)\right]+\text{noise}\; \end{array}$$

in which **x** denotes the coordinates of any point on the frontal thoracic skin, and *α*(*τ*) and *ψ*(*τ*) are time-dependent scalars. At the left side of the “equals” sign (“=”) in eq.(1) the in-vivo voltage *change* is defined as a subtraction, viz. the voltage at point **x** at time *τ*during a specific cardiac cycle *minus* the voltage at the same point **x** at the beginning of that same particular cardiac cycle (*τ*=0).

At each separate time *τ*during a specific cardiac cycle, the in-vivo measured voltage *change* at the left side of eq.(1) is a different linear combination of the standardized (time-independent) *ex-vivo post-mortem* spatial atrial and ventricular fingerprints. The time-dependency is represented only by the coefficients *α*(*τ*) and *ψ*(*τ*), representing the contributions from the atrial changes and the ventricular changes, respectively, to the total voltage change. The key point in our method is that *α*(*τ*) is a measure of atrial volume change, and *ψ*(*τ*)is a measure of ventricular volume change (as will be shown in section Principle of Operation), at that particular instant *τ* during the cardiac cycle. Therefore, the graph of *ψ*(*τ*)as function of time during one single specific heartbeat represents the in-vivo ventricular volume-time curve during that single specific heartbeat in a patient or volunteer. In this paper we will show that, if a sufficiently large number of voltage measuring electrodes is distributed over the thorax, and if the standardized, time-independent *ex-vivo post-mortem* atrial and ventricular fingerprints are known, then the values of the coefficient *ψ*(*τ*) (during any cardiac cycle) during an *in-vivo* measurement on a human volunteer, can be retrieved in a reliable manner on basis of the *in-vivo* measurement data at the left side of eq. (1), i.e. on basis of the voltage changes measured in-vivo on the thoracic skin of the volunteer. In other words, the in-vivo volume-time curve *ψ*(*τ*) of a specific cardiac cycle of a patient can be solved from eq. (1), using the voltage changes measured in-vivo on the thoracic skin of that patient, in combination with the standardized, generic, time-independent *ex-vivo post-mortem* atrial and ventricular fingerprints at the right side of eq. (1).

In our prototype, the Hemodynamic Cardiac Profiler (HCP, TM, pat. pend., [14]), we used seven thoracic voltage measuring electrodes distributed over the thoracic skin, combined into one single compound electrode sticker. These seven electrodes measured six independent voltage differences simultaneously. Our method is *not* based on impedance cardiography (ICG) algorithms, since we do not calculate any impedance measure, but use spatial patterns to retrieve volume changes of specifically the ventricles. Once the volume-time curve *ψ*(*τ*) of a specific cardiac cycle is known, the ventricular stroke volume (SV) of that specific cardiac cycle is known as well, because the stroke volume is calculated as the difference between the end-diastolic ventricular volume at the beginning of that particular cardiac cycle and the end-systolic ventricular volume of that cycle. Therefore, trend curves can be produced, consisting of large series of SV values of subsequent cardiac cycles, representing the behavior of the SV over the course of minutes or hours.

## Principle of Operation

In this section, it is explained (in subsection relative differential measurement) that the positions of the measuring electrodes and their connections to the voltage measuring electronics can be designed in such a way that the unwanted effects of other factors, such as e.g. the velocity-dependency of the conductivity of blood, or changes in the aortic diameter during the cardiac cycle, are minimized.

Subsequently, in subsection algorithm for retrieving ventricular volume-time curves and stroke volume, it is explained how the *ψ*(*τ*) curve can be solved from eq.(1) on basis of the measured voltage change data in combination with the standardized (time-independent) *ex-vivo* spatial atrial and ventricular fingerprints.

### Relative differential measurement

For every point in time during each cardiac cycle, it is to be established (by an algorithm) how much of the measured voltage differences is caused by volume changes in the atria, and how much by volume changes in the ventricles.

The design of the multi-electrode configuration needed to be optimized for this task. Therefore, we formulated the following two demands, which were to be met by the multi-electrode configuration: 

 (i) insensitivity to conductivity changes or diameter changes, during each single cardiac cycle, within vertical elongated structures like the aorta or other larger blood vessels

 (ii) insensitivity to thorax dimensions and other factors that influence the average, global, current strength inside the thorax.

**Figure 1:**
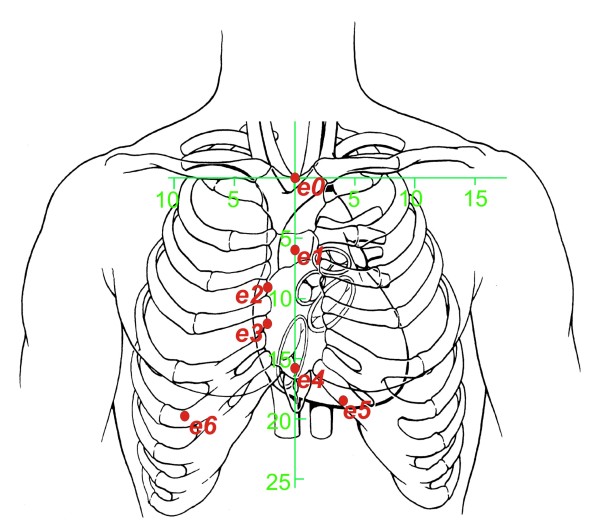
**Schematic rendering the electrode positions with respect to the anatomy of the human thorax.** Distances printed along the green horizontal and vertical axes are in cm. In order to meet the demands (i) and (ii) in subsection relative differential measurement, the electrodes are distributed along the central vertical axis of the sternum. Furthermore, the electrodes spread out to the left and the right side of the thorax *below* the inframammary fold to enable application on female patients without disturbance from the mammae. The positions on the thoracic skin are defined using the green 2D coordinate system (x,z), of which the origin (*x*=0 and *z*=0) coincides with the position of the top end of the sternal bone, near the incisura jugularis. The measuring electrodes are numbered *e*_0_to *e*_6_, and, for each value of *k* (with *k*∈{1…6}), the input voltage $\Phi_{\mathrm{meas}}^{\left(k\right)}$ from eq.(2) is measured between the two electrodes *e*_*k*_and *e*_*k*−1_. The positions of the seven electrodes *e*_0_to *e*_6_are (in (x,z)-coordinates and in mm): (0,0),(0,−60),(−30,−90),(−30,−120),(0,−157),(35,−187), and (−92,−197), respectively. During the tests of the HCP on human volunteers, a self-adhesive “compound electrode sticker” of comfortable and flexible material, containing all measuring electrodes, has been used.

Demand (i) is met if the electrodes are placed in a vertical array over the central sternal line, and the voltages are measured between two adjacent electrodes with a relatively small inter-electrode distance. See the first five measuring electrodes (*e0* to *e4*) in Figure 1. (The other electrodes, *e5* and *e6*, are placed to the left and the right of the vertical sternal line, and used to deal with inter-patient anatomical differences, as explained later on). The measuring electrodes are numbered *e*_0_ to *e*_6_, and, for each value of *k* (with *k*∈{1…6}), the voltage $\Phi_{\mathrm{meas}}^{\left(k\right)}$ is measured between the two electrodes *e*_*k*_and *e*_*k*−1_. This results in a measurement of an approximation of a vertical spatial “differential” of the voltage distribution on the thoracic skin along the vertical sternal line. This “differential” along the vertical axis causes the measured voltage to be relatively insensitive to conductivity and diameter changes that are spread out in the vertical direction, as is the case with large vertical blood vessels.

As has been indicated in the introduction, the basis of the input of our system is formed by the voltage *changes* during a specific cardiac cycle, as function of the time *τ*(in milliseconds), with respect to the beginning of that cardiac cycle at *τ*=0, in which the beginning of a cardiac cycle (*τ*= 0) is defined as the moment in time when the R-peak of the ECG has its maximum. The measured voltage change between electrode pair *k*, as function of *τ*, therefore reads: $\Phi_{\mathrm{meas}}^{\left(k\right)}(\tau )-\Phi_{\mathrm{meas}}^{\left(k\right)}(0)$.

Demand (ii) is met if subsequently this $\Phi_{\mathrm{meas}}^{\left(k\right)}(\tau )-\Phi_{\mathrm{meas}}^{\left(k\right)}(0)$ is divided by $\Phi_{\mathrm{meas}}^{\left(k\right)}(0)$ to form a dimensionless number *φ*_*meas*_:

(2) $$\varphi_{\mathrm{meas}}^{\left(k\right)}(\tau )\;=\;\frac{\Phi_{\mathrm{meas}}^{\left(k\right)}(\tau )\;-\;\Phi_{\mathrm{meas}}^{\left(k\right)}(0)}{\Phi_{\mathrm{meas}}^{\left(k\right)}(0)}$$

As will be shown in the Appendix, the voltage change $\Phi_{\mathrm{meas}}^{\left(k\right)}(\tau )-\Phi_{\mathrm{meas}}^{\left(k\right)}(0)$ is proportional to the strength of the incident applied current field near the heart. Furthermore, the $\Phi_{\mathrm{meas}}^{\left(k\right)}$ itself is proportional to the incident applied current field (near electrodes *e*_*k*_and *e*_*k*−1_), for all values of *τ*(including *τ*=0).

Therefore, the strength of the applied current is factored out due to the quotient in eq.(2), and the dimensionless numbers $\varphi_{\mathrm{meas}}^{\left(k\right)}(\tau )$ are insensitive to variations in applied global current density.

### Algorithm for retrieving ventricular volume-time curves and stroke volume

In *ex-vivo* calibration experiments on human postmortem hearts (to be described in more detail in section Methods: ex-vivo post-mortem calibration), we measured $\Phi_{\mathrm{ventric},\mathrm{before}}^{\mathrm{postmortem}}$ and $\Phi_{\mathrm{ventric},\mathrm{after}}^{\mathrm{postmortem}}$ values on a post-mortem thorax, in which $\Phi_{\mathrm{ventric},\mathrm{after}}^{\mathrm{postmortem}}$ is measured after an artificial increase of the volume (with a known fixed standardized volume ${\tilde{V}}_{\mathrm{calib}}$) in exclusively the ventricles of the post-mortem heart, and $\Phi_{\mathrm{ventric},\mathrm{before}}^{\mathrm{postmortem}}$ is the initial value before the artificial increase.

Completely analogous to the definition of $\varphi_{\mathrm{meas}}^{\left(k\right)}$ in eq.(2), the $\Phi_{\mathrm{ventric},\mathrm{before}}^{(k),\mathrm{postmortem}}$ and $\Phi_{\mathrm{ventric},\mathrm{after}}^{(k),\mathrm{postmortem}}$ values are combined into a dimensionless scalar $\mu_{\mathrm{ventric}}^{(k),\mathrm{postmortem}}$:

(3) $$\mu_{\mathrm{ventric}}^{(k),\mathrm{postmortem}}=\frac{\Phi_{\mathrm{ventric},\mathrm{after}}^{(k),\mathrm{postmortem}}-\Phi_{\mathrm{ventric},\mathrm{before}}^{(k),\mathrm{postmortem}}}{\Phi_{\mathrm{ventric},\mathrm{before}}^{(k),\mathrm{postmortem}}}$$

in which the index (*k*) refers to exactly the same locations on the thorax as the positions of the electrodes *e*_*k*_ in the *in-vivo* situation in eq.(2).

These six $\mu_{\mathrm{ventric}}^{(k),\mathrm{postmortem}}$ scalars are combined into a 6-dimensional “ventricular fingerprint vector” **f**_*V*_:

(4) $$f_{V}\;=\;{\left\{\;\mu_{\mathrm{ventric}}^{(1),\mathrm{postmortem}},\mu_{\mathrm{ventric}}^{(2),\mathrm{postmortem}},\cdots \;,\mu_{\mathrm{ventric}}^{(6),\mathrm{postmortem}}\;\right\}}^{T}$$

(in which T indicates the transpose, switching between the horizontal and vertical notation of a vector). Analogously, for ex-vivo post-mortem measurements of artificial filling of only the atria, we have the 6-dimensional “atrial fingerprint vector” **f**_*A*_:

(5) $$f_{A}\;=\;{\left\{\;\mu_{\mathrm{atrial}}^{(1),\mathrm{postmortem}},\mu_{\mathrm{atrial}}^{(2),\mathrm{postmortem}},\cdots \;,\mu_{\mathrm{atrial}}^{(6),\mathrm{postmortem}}\;\right\}}^{T}$$

Likewise, the *φ*_*meas*_values that are measured *in-vivo* on a patient (see eq.(2)), are combined into a 6-dimensional measurement vector **v**_*meas*_:

(6) $$v_{\mathrm{meas}}(\tau )\;=\;{\left\{\;\varphi_{\mathrm{meas}}^{\left(1\right)}(\tau ),\varphi_{\mathrm{meas}}^{\left(2\right)}(\tau ),\cdots \;,\varphi_{\mathrm{meas}}^{\left(6\right)}(\tau )\;\right\}}^{T}$$

As has been indicated in the introduction using eq.(1), the *φ*_*meas*_values, measured in-vivo on a patient, are considered to be a *linear combination* of the effects from the standard atrial ex-vivo post-mortem volume change (fingerprint vector **f**_*A*_), and the standardized ventricular ex-vivo post-mortem volume change (fingerprint vector **f**_*V*_). The coefficients in this linear combination are the scalars *α*(*τ*)and *ψ*(*τ*):

(7) $$\left[\begin{array}{l} \varphi_{\mathrm{meas}}^{\left(1\right)}(\tau )\\ \varphi_{\mathrm{meas}}^{\left(2\right)}(\tau )\\ \cdots \\ \varphi_{\mathrm{meas}}^{\left(6\right)}(\tau ) \end{array}\right]\;=\;\alpha (\tau )\left[\begin{array}{l} \mu_{\mathrm{atrial}}^{(1),\mathrm{postmortem}}\\ \mu_{\mathrm{atrial}}^{(2),\mathrm{postmortem}}\\ \cdots \\ \mu_{\mathrm{atrial}}^{(6),\mathrm{postmortem}} \end{array}\right]\;+\;\psi (\tau )\left[\begin{array}{l} \mu_{\mathrm{ventric}}^{(1),\mathrm{postmortem}}\\ \mu_{\mathrm{ventric}}^{(2),\mathrm{postmortem}}\\ \cdots \\ \mu_{\mathrm{ventric}}^{(6),\mathrm{postmortem}} \end{array}\right]\;+\;\left[\begin{array}{l} \mathrm{noise}\\ \mathrm{noise}\\ \cdots \\ \mathrm{noise} \end{array}\right]$$

or, equivalently,

$$v_{\mathrm{meas}}(\tau )\;=\;\alpha (\tau )f_{A}\;+\;\psi (\tau )f_{V}\;+\;\mathrm{noise}$$

In order to retrieve the stroke volume and volume-time curves from the measured **v**_*meas*_(*τ*), the HCP has to solve the value of *ψ*(*τ*)from eq.(7) for each point in time *τ*at which a measurement of **v**_*meas*_has taken place (every 5 milliseconds in our HCP prototype).

The last equation (eq. (7)), together with the method to solve the volume-time curves (*ψ*(*τ*)) from eq. (7) using the measured **v**_*meas*_(*τ*), constitute the core of the new VFR method presented in this paper.

Therefore, equation (7) has to be accounted for in the form of a fundamental physical reasoning that justifies this equation. This reasoning is provided in the Appendix (A1).

Furthermore, fundamentally speaking, any calculation of voltage changes on basis of volume changes of blood-filled lumina such as the ventricles, is a *non-linear* problem. Therefore, the limits of the accuracy of this linear equation will be examined in the Appendix as well (in A2).

A method for retrieving *ψ*(*τ*)from equation 7 is described directly below.

Given the possible presence of noise and artefacts, which are generally unrelated to the fingerprint patterns, the task for the real-time algorithm is to find the parameters *α*(*τ*)and *ψ*(*τ*) for which

(8) $$|v_{\mathrm{meas}}(\tau )\;-\;\left(\alpha (\tau )f_{A}+\psi (\tau )f_{V}\right)|\;\;\text{is minimal}$$

This task is performed by finding the *α*(*τ*)and *ψ*(*τ*) that satisfy:

(9) $$\;(v_{\mathrm{meas}}(\tau )\;-\;(\alpha (\tau )f_{A}+\psi (\tau )f_{V}))\;\cdot \;f_{A}\;=\;0\;\;\wedge \;\;(v_{\mathrm{meas}}(\tau )\;-\;(\alpha (\tau )f_{A}+\psi (\tau )f_{V}))\;\cdot \;f_{V}\;=\;0$$

in which the inner product (“·”) between any two 6D-vectors **A** and **B** is defined completely analogously to the familiar 3-dimensional case, i.e. $A\;\cdot \;B=\overset{6}{\underset{i=1}{\sum }}A_{i}B_{i}$. Using linear algebra, eq.(9) is easily solved, e.g. by multiplying the equation at the left of the logical “AND” symbol in eq.(9) with (**f**_*A*_·**f**_*V*_), and multiplying the equation at the right of the logical “AND” symbol with (**f**_*A*_·**f**_*A*_), and subsequently subtracting the two resulting equations, which eliminates the variable *α*(*τ*). The solution then reads

(10) $$\alpha (\tau )\;=\;W_{A}\;\cdot \;v_{\mathrm{meas}}(\tau )\;\;\;\text{and}\;\;\;\psi (\tau )\;=\;W_{V}\;\cdot \;v_{\mathrm{meas}}(\tau )$$

in which

(11) $$\begin{array}{lll} W_{A}\;= & \;\frac{(f_{V}\;\cdot \;f_{V})f_{A}\;\;-\;\;(f_{A}\;\cdot \;f_{V})f_{V}}{(f_{A}\;\cdot \;f_{A})(f_{V}\;\cdot \;f_{V})\;\;-\;\;{\left(f_{A}\;\cdot \;f_{V}\right)}^{2}}\; & \;\\ W_{V}\;= & \;\frac{(f_{A}\;\cdot \;f_{A})f_{V}\;\;-\;\;(f_{V}\;\cdot \;f_{A})f_{A}}{(f_{A}\;\cdot \;f_{A})(f_{V}\;\cdot \;f_{V})\;\;-\;\;{\left(f_{A}\;\cdot \;f_{V}\right)}^{2}}\; \end{array}$$

The “weight vectors” **W**_*A*_and **W**_*V*_are time-independent and completely based on only the ex-vivo “fingerprint” vectors **f**_*A*_ and **f**_*V*_. As a result, the *ψ*(*τ*)is a linear function of the six measured voltages in the **v**_*meas*_(*τ*)vector in eq.(10).

On basis of the results of the ex-vivo post-mortem calibration experiments (that will be described in section Methods: ex-vivo post-mortem calibration), a fixed known standard ventricular volume increase ${\tilde{V}}_{\mathrm{calib}}$ has been linked to the “fingerprint vector” **f**_*V*_. Therefore, as an extreme and purely theoretical example, consider a case in which the volume of the atria would not change at all, and in which at some moment in time *τ*, the **v**_*meas*_(*τ*) would be equal to **f**_*V*_. Then the value of *ψ*(*τ*)would be equal to one (as can be seen in eq.(7)), and the ventricular volume change *V*(*τ*)−*V*(0) (in which *V*(*τ*)denotes the ventricular volume at time *τ*during a specific cardiac cycle, and *V*(0)denotes the ventricular volume at the beginning (*τ*=0) of that specific cardiac cycle) would be equal to ${\tilde{V}}_{\mathrm{calib}}$ at time *τ*.

In a realistic situation, in which the atrial and ventricular volumes change simultaneously, we have (on basis of eq.(10)):

(12) $$V(\tau )\;-\;V(0)\;=\;\psi (\tau )\;{\tilde{V}}_{\mathrm{calib}}\;=\;(v_{\mathrm{meas}}(\tau )\;\cdot \;W_{V})\;\;{\tilde{V}}_{\mathrm{calib}}$$

This equation constitutes the final result that has been implemented in the algorithm of the experimental prototype of the HCP, in order to calculate the ventricular volume-time curve *V*(*τ*)−*V*(0)from the **v**_*meas*_(*τ*)measured in-vivo on volunteers.

## Methods: ex-vivo post-mortem calibration

In order to perform realistic measurements ex-vivo, the front part of a post-mortem human thorax conserved in formalin was placed in a basin filled with a NaCl solution.

The concentration of the NaCl in the solution was such that the solution had a specific conductivity equal to the average conduction of thoracic tissue. The thorax was suspended halfway into the solution in such a way that the thoracic skin remained dry, but the rest of the thorax, including the curved inner side of the thoracic wall, was in direct contact with the NaCl solution.

**Figure 2:**
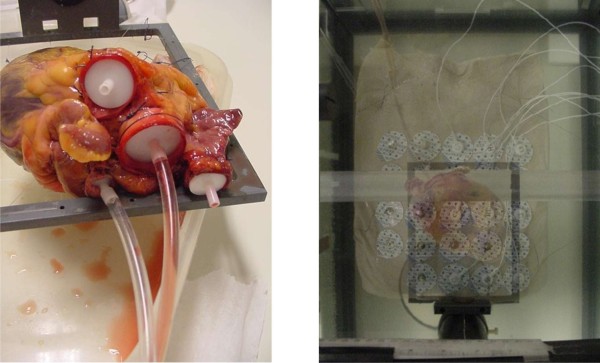
***left:* Post-mortem human heart, with connecting tubes for the purpose of producing well-defined volume changes during the ex-vivo post-mortem calibration measurements as described in section Methods: ex-vivo post-mortem calibration.***right:* Experimental set-up with a postmortem human thorax, a post-mortem human heart, and a matrix of 5 ×5 measuring electrodes on the skin of the postmortem human thorax. For visibility purposes, the thorax has been made semi-transparent in this image, in order to show the position of the heart lying underneath.

On the dry thoracic skin, a matrix of measuring electrodes was placed, in a two-dimensional array of 5 ×5 electrodes, with an interelectrode distance (as measured from the center of one electrode to the center of the next) of 35 mm in both the vertical and horizontal direction (see Figure 2). Each individual electrode from this 5 × 5 matrix was connected to a voltage measuring device, together with a reference measuring electrode that was placed centrally on the cranial ending of the sternum, in the middle of the incisura jugularis.

Furthermore, a human post-mortem heart was suspended directly below the post-mortem human thorax using a hard-plastic frame with a grid of fishing wire. In order to be able to perform measurements during filling of *only* the ventricles (or, c.q., *only* the atria), it was essential that all heart valves within the heart were closed. Because of the friability of the leaflets of the valves, patches of thin tissue were sutured over the orifice of the valves. The coronary arteries were ligatured at the root. In some cases, cyanoacrylate was used to seal a few small remaining leaks.

Measurements of the voltage field were made using the 2D electrode matrix, during filling and emptying of the different compartments of the heart. These compartments (atria and ventricles) were connected to plastic tubing and syringes (see left side of Figure 2) to enable controlled filling and emptying with a blood-equivalent NaCl solution. Plastic wire-straps were used to keep the tubing absolutely fixed and immobilized during the experiments. In order to let the heart retain a realistic shape during volume change of the ventricles, a simultaneous movement of two separate syringes was performed, filling the ventricles with 50 ml of blood-equivalent NaCl solution (having the same specific electric conductivity as blood), and simultaneously removing 50 ml of thorax-equivalent solution (i.e. a solution containing less NaCl, having the same average specific electric conductivity as the inside of the thorax) from the atria. This procedure caused the total heart volume to remain invariant. Furthermore, since the specific conductivity of the solution that was removed from the atria was equal to that of the solution outside the heart (viz. the thorax-equivalent solution), the electric effect of the volume decrease in the atria was annulled. At the same time however the electric effect of the volume decrease in the *ventricles* was *not* annulled because the specific conductivity of the removed blood-equivalent solution was much higher than that of the thorax-equivalent solution.

From initial experimental measurements in-vivo, we have learned already in an early stage of the development, that stable measurements in-vivo were obtained only if the measuring electrodes were placed on skin that covered the thoracic chest, i.e. unstable measurements resulted if the electrodes were placed on skin covering the abdomen. Since in the post-mortem ex-vivo measurements a post-mortem thorax was used, the post-mortem ex-vivo measurements were confined to the thoracic skin as well. No measurements were done ex-vivo on locations on the post-mortem skin that would have no in-vivo counterpart in the form of measurements on the same location on the skin in-vivo.

## Methods: in-vivo tests of the HCP on volunteers

### Experimental prototype of HCP

We developed a measuring device that consists of eight separate units, each containing a current source and an amplifier-demodulator [17,18]. In this study, only six of these units were used. Each current source produces an alternating current of 1 mA_*pp*_ (milliAmperes peak-to-peak) at a distinct frequency in the range between 58 kHz and 90 kHz. The current sources are added so as to develop an electric field in the thorax containing components of different frequencies each to be measured by the according amplifier- demodulator. The current sources were located at approximately 2 meters from the volunteers in a patient-safe metal “front-end” rack system. The maximum applied current will not exceed 8 mA_*pp*_ which is well within the limits determined by the standard IEC-601-1, sub-clause 8.7.3. Each measuring channel contains an ECG amplifier that simultaneously detects the ECG as presented at the electrodes for measurement of the electric field that is developed by the injected current. In this manner 12 outputs are available: 6 outputs related to the voltages at a certain site on the thorax as a result of the injected currents and another 6 outputs presenting the ECG at this site. Moreover, each output containing data from the field developed by the injected current is split into two frequency ranges: a low-pass section and a band-pass section. The low-pass section with a cut-off frequency of 0.072 Hz produces an average voltage, *V*_*avg*_, whereas the band-pass section produces an output in a bandwidth from 0.072 Hz up to 34 Hz. The latter output is amplified giving a voltage, *V*_*fluct*_, rendering the instantaneous fluctuations in V with respect to the basic level *V*_*avg*_. The current source and input amplifier of each unit are properly isolated according to the standards for body floating application to ensure safe operation when applied on patients. All data is digitized at a sampling rate of 200 Hz and made available through a serial communication interface that leads to a personal computer (PC). The system is equipped with two PC’s. During a time interval of exactly 20 seconds (a 20 seconds “measurement period”), PC #1 collected real time voltage data from the filter/amplifier unit. Directly after this interval of 20 seconds, there is a short interval of 4.3 seconds during which PC #1 collects no input data, but sends the compressed data buffered from the last “measurement period” on to PC #2. Immediately after that, PC #1 starts with the next “measurement period” of 20 seconds, during which PC #2 processes the data it just received from PC #1, i.e. PC #2 applies the VFR algorithm and displays the resulting ventricular volume-time curves, as well as the average Stroke Volume associated with the last measurement period of 20 seconds. The time that PC #2 needed for applying the algorithm and displaying the result was 14.8 seconds. Therefore, the “repetition time” of the total system (i.e., the time that lapsed between two subsequent updates of the displayed Stroke Volume) was 24.3 (= 20 + 4.3) seconds. Furthermore, the maximum delay between actual measurement of data (at the beginning of a measurement period of 20 seconds) to displaying the results is 39.1 (= 20 + 4.3+ 14.8) seconds.

### Respiration-gated algorithm

In order to exclude artefacts in the measured voltage change pattern ($\varphi_{\mathrm{meas}}^{\left(k\right)}$ with *k*∈{1,2,…,6}) on the thoracic skin due to distortions caused by changing air volumes inside the lungs, we adopted a respiration-gated approach. Preliminary measurements have shown that the absolute value of $\Phi_{\mathrm{meas}}^{\left(k\right)}$ (as opposed to the dimensionless relative difference $\varphi_{\mathrm{meas}}^{\left(k\right)}$ ) is a reliable indicator of the volume of air inside the lungs. Therefore, by monitoring the respiratory fluctuation in the series of absolute values of *Φ*_*meas*_(0)from subsequent heart cycles, the HCP automatically selects a subset of cardiac cycles, out of the total set of cardiac cycles within a period of 20 seconds, that corresponds to a specific phase in the respiratory cycle (on basis of the trend in the series of $\Phi_{\mathrm{meas}}^{\left(k\right)}(0)$ values), as well as to the same volume of air inside the lungs (on basis of the absolute value of $\Phi_{\mathrm{meas}}^{\left(k\right)}(0)$ values). In this way, this “*Φ*-gated” algorithm serves as a respiration-gated algorithm. This selection procedure on basis of the $\Phi_{\mathrm{meas}}^{\left(k\right)}(0)$ values takes place *before* the algorithm (as specified in eq.(12)) is applied.

### G-suit deflation, and LVot Doppler as a reference technique

In order to cause changes in stroke volume in volunteers, and at the same time let other (environmental) parameters remain the same as much as possible, we used inflatable antishock trousers (known as “G-suits” to air force pilots). Such antishock trousers contain inflatable bladders inside the trousers. When inflated, these bladders produce pressure forces onto the legs, which prevent the blood to accumulate inside the legs when the airplane makes a sharp turn; hence these trousers prevent the pilot from loosing consciousness. It has been shown [19] that, in a standing position, sudden deflation of an inflated G-suit can cause the stroke volume to drop suddenly. We performed a procedure on 7 volunteers, in which a G-suit (Anti-G-Garment, CUTAWAY, CSU-13B/P, USAF, USA) was inflated gradually from 0 mmHg to 70 mmHg during 2 minutes and 30 seconds. Subsequently, a constant pressure of 70 mmHg pressure was maintained during 2 minutes, after which, finally, a sudden deflation back to 0 mmHg took place within 5 seconds.

The 7 volunteers were all healthy males, with a Body Mass Index ranging from normal to slightly overweight. The volunteers had no visible abnormalities in their thoracic anatomy, and were randomly selected from the male population on the Utrecht University Campus. All volunteers have read and signed an Informed Consent form, prior to the experiments. All procedures concerning the volunteers in this study have been executed in compliance with the guidelines of the University Medical Center Utrecht (and in compliance with the Helsinki Declaration), and have been approved by the Authority for Safety of the University Medical Center Utrecht.

Simultaneously, during the entire procedure, continuous HCP recordings, as well as LVot (Left Ventricular outflow tract) Doppler recordings were performed, using a Philips CX50 echocardiograph (version 2.0, Philips Medical Systems, Eindhoven, The Netherlands) with a S5-1 ultrasound transducer. Stroke volume was calculated as the Velocity Time Integral (VTI) multiplied by the LVot area. Pulse contour analysis was done offline for Velocity Time Integral (VTI). VTI was traced by hand and calculated by the echocardiograph on 1-3 curves in the each LVot registration. LVot registrations where made sequential with intervals of 30 sec. at rest, 20 sec during inflation-inflated and 3-4 sec. during deflation. Each registration holds 3 to 5 flow curves depending on the volunteer’s heart rate. In the apical 5-chamber view the Pulsed Wave Doppler sample volume was positioned about 0.5 cm proximal of the Aorta-valve in line with the blood flow direction as indicated by the color Doppler. This position was regularly checked, depending on the movements of the volunteer. The LVot area was calculated using the measured LVot diameter, average of 2 measurements, in a enlarged view of the Aorta-valve in the para-sternal long axes view.

This validation focussed on investigating the capability of monitoring changes in SV during de G-suit manipulations correctly. For each individual volunteer, a single and constant scaling factor *K*_*volunteer*_ was applied to the SV readings to let the average initial SV from the HCP coincide with the SV from the LVot Doppler.

## Results

### results of the ex-vivo post-mortem calibration

For each of the electrodes in the electrode matrix shown in Figure 2, the voltages $\Phi_{\mathrm{ventric},\mathrm{before}}^{\mathrm{postmortem}}$ and $\Phi_{\mathrm{ventric},\mathrm{after}}^{\mathrm{postmortem}}$ have been measured, as well as the voltages $\Phi_{\mathrm{atrial},\mathrm{before}}^{\mathrm{postmortem}}$ and $\Phi_{\mathrm{atrial},\mathrm{after}}^{\mathrm{postmortem}}$, in which “before” and “after” refer to the moment in time with respect to the artificial filling procedure. All voltages were measured with respect to a reference electrode at the incisura jugularis (not shown in the figures). Two-dimensional linear interpolation has been used to create the continuous two-dimensional interpolations $\Phi_{\mathrm{ventric},\mathrm{before}}^{\mathrm{postmortem}}(x,z)$ and $\Phi_{\mathrm{ventric},\mathrm{after}}^{\mathrm{postmortem}}(x,z)$, as well as $\Phi_{\mathrm{atrial},\mathrm{before}}^{\mathrm{postmortem}}(x,z)$ and $\Phi_{\mathrm{atrial},\mathrm{after}}^{\mathrm{postmortem}}(x,z)$ from the discrete sets of measurements from the electrode matrix, in which the origin (x=0 and z=0) coincides with the position of the top end of the sternal bone, near the incisura jugularis, and *x* and *z* denote the horizontal and vertical position with respect to the origin, respectively.

Whereas in eq.3 the *μ*values were defined only for the 7 positions on the thorax on which the 7 electrodes (*e*_0_ to *e*_6_) were located, we now define a similar value for each point (x,z) on the thoracic skin, in complete analogy to eq.3, using the above mentioned, experimentally obtained, interpolations $\Phi_{\mathrm{ventric},\mathrm{before}}^{\mathrm{postmortem}}(x,z)$, $\Phi_{\mathrm{ventric},\mathrm{after}}^{\mathrm{postmortem}}(x,z)$, $\Phi_{\mathrm{atrial},\mathrm{before}}^{\mathrm{postmortem}}(x,z)$ and $\Phi_{\mathrm{atrial},\mathrm{after}}^{\mathrm{postmortem}}(x,z)$ :

(13) $$m_{\mathrm{ventric}}^{\mathrm{postmortem}}(x,z)=\frac{\Phi_{\mathrm{ventric},\mathrm{after}}^{\mathrm{postmortem}}(x,z)-\Phi_{\mathrm{ventric},\mathrm{before}}^{\mathrm{postmortem}}(x,z)}{\Phi_{\mathrm{ventric},\mathrm{before}}^{\mathrm{postmortem}}(x,z-15)-\Phi_{\mathrm{ventric},\mathrm{before}}^{\mathrm{postmortem}}(x,z+15)}$$

and

(14) $$m_{\mathrm{atrial}}^{\mathrm{postmortem}}(x,z)=\frac{\Phi_{\mathrm{atrial},\mathrm{after}}^{\mathrm{postmortem}}(x,z)-\Phi_{\mathrm{atrial},\mathrm{before}}^{\mathrm{postmortem}}(x,z)}{\Phi_{\mathrm{atrial},\mathrm{before}}^{\mathrm{postmortem}}(x,z-15)-\Phi_{\mathrm{atrial},\mathrm{before}}^{\mathrm{postmortem}}(x,z+15)}$$

**Figure 3:**
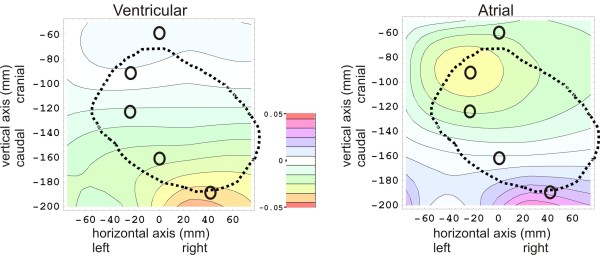
**Results of the *ex-vivo post-mortem* calibration measurements, showing a ventricular ’fingerprint’ plot at the left, an atrial ’fingerprint’ plot at the right, and a color scale legend in the middle.** The horizontal and vertical axis in each plot represents the horizontal position *x* (in mm) and the vertical position *z* (in mm) on the thoracic skin, respectively, in which the origin (*x*=0 and *z*=0) coincides with the position of the top end of the sternal bone, near the incisura jugularis. In both fingerprint plots, the *m*(*x*,*z*)values are plotted as a color for each 2D position (x,z) on the thoracic skin. (See eqs. (13) and (14) for the definition of *m*(*x*,*z*)). The thick dotted black line in both plots denotes the contour of the heart lying below the thoracic skin. The positions of five electrodes (*e*_1_to *e*_5_)are indicated by black O-shaped symbols. In the plot on the *left*, the ventricular ’fingerprint’ is rendered, i.e. results are rendered of filling the ventricles with blood-equivalent solution, and simultaneously removing thorax-equivalent solution from the atria. In the plot on the *right*, the atria are filled with blood-equivalent solution, and thorax-equivalent solution is removed from the ventricles simultaneously.

The 2D color contour plots of $m_{\mathrm{ventric}}^{\mathrm{postmortem}}(x,z)$ and $m_{\mathrm{atrial}}^{\mathrm{postmortem}}(x,z)$ are rendered at the left side and the right side of Figure 3, respectively.

The positions of five electrodes (*e*_1_to *e*_5_) are indicated in Figure 3 by small O-shaped symbols.

The “+15” and “-15” in the denominator of eqs. (13) and (14) indicate that the normalization of the m-values in these plots are based on the inter-electrode distances (in the vertical direction) between *e*_1_, *e*_2_, and *e*_3_ (viz: 30 mm).

The $\mu_{\mathrm{ventric}}^{(k),\mathrm{postmortem}}$ are now calculated easily as:

(15) $$\mu_{\mathrm{ventric}}^{(k),\mathrm{postmortem}}=m_{\mathrm{ventric}}^{\mathrm{postmortem}}(\text{position of}e_{k})-m_{\mathrm{ventric}}^{\mathrm{postmortem}}(\text{position of}e_{k-1})$$

for (k) ∈{2,3}.

The $\mu_{\mathrm{ventric}}^{(k),\mathrm{postmortem}}$ of the other electrode pairs are calculated analogously, taking into account other inter-electrode distances. The $\mu_{\mathrm{atrial}}^{(k),\mathrm{postmortem}}$ are calculated in the same way, on basis of the $m_{\mathrm{atrial}}^{\mathrm{postmortem}}(x,z)$ in eq.(14).

### Results of the in-vivo tests of the HCP on human volunteers

**Figure 4:**
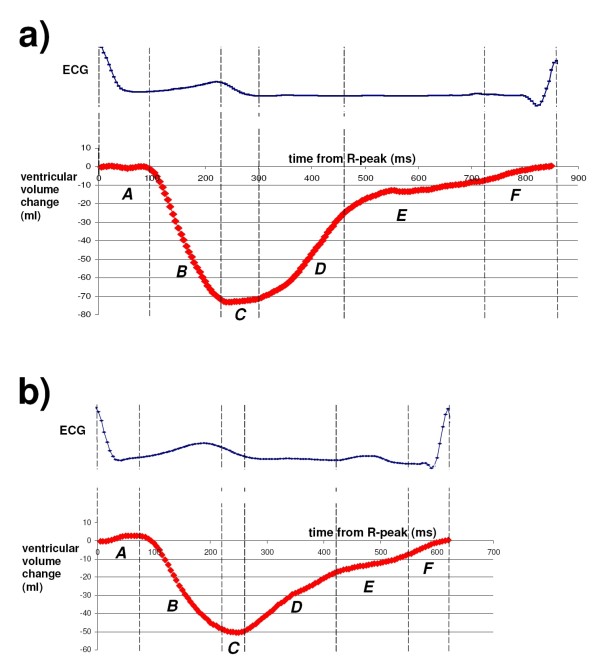
**Typical examples of a ventricular volume-time curves (red, thick line), produced by the HCP.** These volume-time curves were measured on human volunteers, in a standing position, *before* inflation of the G-suit. The vertical axis shows the ventricular volume change (*V*(*τ*)−*V*(0)) in ml, as calculated using eq. 12; the horizontal axis shows the time *τ*(i.e., the time that has lapsed since the last R-peak of the ECG), in milliseconds. ***a:*** volunteer with a heart rate of 69 bpm, stroke volume of 73 ml, and cardiac output of 5.0 l/min. ***b:*** volunteer with a higher heart rate of 94 bpm, stroke volume of 50 ml, and cardiac output of 4.7 l/min. In both (**a**) and (**b**), the ECG (thin line, blue) was measured simultaneously by the HCP, and has been plotted above the corresponding volume-time plot. For each of the volunteers, five different phases of the cardiac cycle (indicated by *A* to *F* in the volume-time graphs) can be recognized: *A* = Isovolumetric contraction phase, *B* = Ejection phase, *C* = Isovolumetric relaxation phase, *D* = Rapid filling phase, *E* = Diastasis, *F* = Atrial kick. For each volume-time plot, the dashed vertical lines indicate the boundaries between the various heart phases.

In Figure 4, typical examples are rendered of ventricular volume-time curves, produced by the HCP, of a volunteer in rest, in a standing position, *before* inflation of the G-suit, in which the “ventricular volume change” (i.e., *V*(*τ*)−*V*(0)) was calculated using eq. 12.

**Figure 5:**
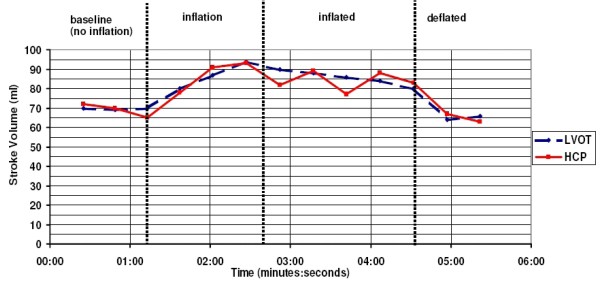
**Example of a plot of the stroke volume as function of time (in minutes), according to the HCP (red, continuous line), and LVot Doppler, during the G-suit experiment.** The three thick dashed vertical black lines denote the following instances (from left to right, respectively): (i) starting of the inflation pump connected to the G-suit, (ii) maximum pressure reached, (iii) opening of the valve that releases the pressurized air from the G-suit immediately. During inflation of the G-suit, the stroke volume increases. Furthermore, directly after the sudden deflation at 4 minutes and 40 seconds, the stroke volume decreases. This is consistent with data from the literature (e.g. [19]).

An example of the simultaneous SV readings from HCP and LVot Doppler, as function of time, during inflation and deflation of the G-suit is rendered in Figure 5.

**Figure 6:**
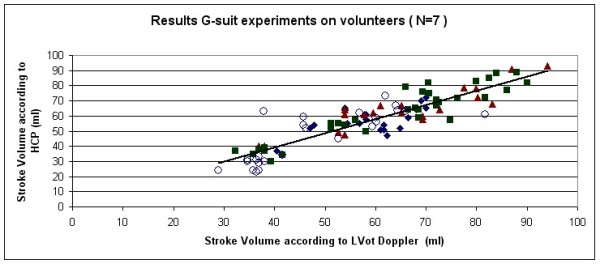
**Results of the G-suit validation experiments on 7 volunteers: plot of the Stroke Volume according to the HCP as function the Stroke Volume according to the LVot Doppler.** Both axes are in ml. The Pearson correlation coefficient was *R*=0.892. Four different marker symbols have been used to indicate the points, in which each symbol corresponds to one of the four stages during the G-suit protocol: Black diamonds = baseline, Brown triangles = inflation, Green squares = inflated, Blue Circles = deflated).

**Figure 7:**
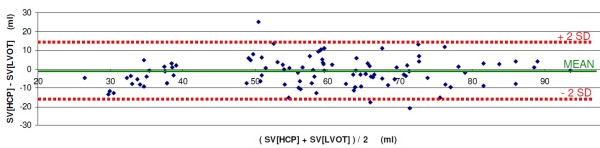
**Bland-Altman plot of the results of the G-suit validation experiments on 7 volunteers.** The mean value (continuous line, green), and the upper and lower limits of agreement (dashed lines, red) are rendered. See text.

The combined results of all 7 volunteers are rendered in Figure 6 (Linear Regression) and Figure 7 (Bland Altman plot).

The Pearson correlation coefficient in Figure 6 was *R*=0.892.

In the Bland-Altman plot, the mean bias was -1.6 ml, with 2SD = 14.8 ml. The upper limit of agreement was 13.2 ml, and the lower limit of agreement was -16.4 ml.

## Discussion

### Ex-vivo post-mortem measurements

The ex-vivo post-mortem measurements show that the spatial 2D distribution of voltage changes over the frontal thoracic skin due to ventricular emptying (the ventricular “fingerprint”) is clearly different from the atrial “fingerprint”.

**Figure 8:**
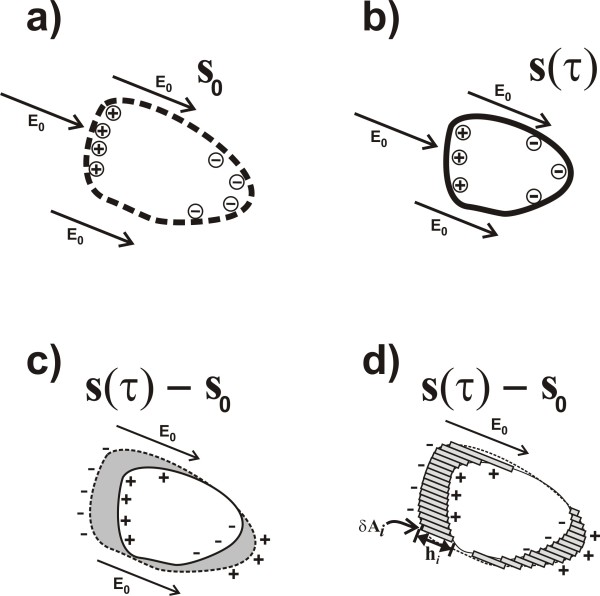
**Schematics illustrating the relation between a volume change of some blood filled lumen (such as e.g. a ventricle) and the sum of dipoles that represent the first order alteration of the E-field due to the volume change, as explained in the first subsection of the Appendix.*a:*** The initial volume of blood (at *τ*=0) is bounded by a closed surface $A_{0}$ (indicated by the dashed line) that has non-zero values of **s**_0_. At each surface element *δ***A**_*i*_of the surface $A_{0}$, there is a charge *q*_*i*_that is proportional to (**E**_0_ · *δ***A**_*i*_). The charges on the boundary surface $A_{0}$ are indicated by the ⊕and ⊖signs. (Evidently, in an applied AC current field, the boundary charges change signs continuously, along with the direction of the arrow of the applied **E**_0_, with the same frequency *f * of the AC current. In this schematic however, only the moments in time are depicted in which the arrows point in one specific direction). ***b:*** Situation at some time *τ*>0during the same cardiac cycle. The volume of blood has become smaller, and is now bounded by the non-zero values of **s**(*τ*). ***c:*** Subtraction of the volumes from (**a**) and (**b**), in which the volume difference *V*(*τ*)−*V*(0)is indicated by the grey shade. If the voltage distributions on the thoracic skin in situation (**a**) and (**b**) are denoted by *Φ*(**x**,0)and *Φ*(**x**,*τ*), respectively, then the voltage change *Φ*(**x**,*τ*)−*Φ*(**x**,0)corresponds to **s**(**x**,*τ*)−**s**_0_(**x**), and hence also corresponds to the dipoles in (**b**) minus the dipoles in (**a**). ***d:*** The grey-shaded volume difference *V*(*τ*)−*V*(0)equals the sum of a set of small rectangular beams : $V(\tau )-V(0)\;=\;\underset{i}{\sum }\;h_{i}\cdot \left(\delta A_{i}\right)$, which is quite similar to the sum of the dipoles formed by the charges at the end-points of the rectangular beams, corresponding to the dipole interpretation of the charges in (**c**).

A number of features can be discerned in the experimental results in Figure 3. In the atrial fingerprint at the right side of Figure 3, a yellow (negative) blob (minimum value of about -0.03) centered around (-30,-70) is visible, as well as a positive counterpart (purple, positive blob, maximum value of about 0.03) near (30, -180). In the Appendix, and more particularly in Figure 8, it was predicted that the normalized voltage change distribution on the thoracic skin (fingerprint) in the post-mortem ex-vivo experiments should have approximately the same appearance as if the heart were replaced by a set of electric dipoles. The presence of the yellow (negative) blob and the purple, positive blob in the atrial plot in Figure 3 are in accordance with a “dipole-like field” from a set of dipoles located inside the heart.

In the ventricular fingerprint (at the left side of Figure 3), it can be seen that the entire “dipole-like field” is positioned much lower (i.e., displaced in the caudal direction with respect to the atrial “dipole” with a distance of about 120 mm). For reasons explained in the Methods section, the post-mortem ex-vivo measurements were confined to the thoracic skin. Therefore, only the upper part of the top blob (orange, negative) was located on the post-mortem thorax in the ex-vivo ventricular fingerprint measurements, at about (30,-180) in the left panel in Figure 3.

### In-vivo tests on human volunteers

The shapes of the ventricular volume-time curves (see Figure 4) were consistent with the volume-time curves from the literature. The realistic shapes of the ventricular volume-time curves produced by the HCP during the tests on human volunteers indicate that the atrial and ventricular fingerprints are indeed different enough to enable the HCP algorithm.

Furthermore, the “relative differential measurement” method, in which electrodes are placed relatively close to each other along the vertical central sternal line (as explained in subsection relative differential measurement), seems to be effective in suppressing the influence of conductivity changes in larger vertical blood vessels inside the thorax. (Or, more precisely: the relative differential measurement method is relatively insensitive to changes in diameter and blood conductivity (which varies with blood velocity) that occur within vertical elongated conductors like the aorta, vena cava, and pulmonary arteries during the cardiac cycle).

One volunteer (volunteer *♯*8) was excluded from the validation section because LVot Doppler incidently detected a leakage of the mitral valve. Interestingly, the HCP has produced a volume-time curve for this volunteer that is distinctly different from the volume-time curves of the other volunteers, and, moreover, this particular volume-time curve was assessed by cardiologists to be typical for mitral valve leakage. Further research is warranted to investigate if the HCP not only can produce reliable stroke volume values, but also may be used to determine particular patterns in the volume-time curves to diagnose e.g. diastolic dysfunction, cardiomyopathy or valvular disease.

Therefore, the ventricular volume-time curves have the potential of being a useful diagnostic monitoring tool as well.

### Comparison of the VFR with other techniques

In contrast to Impedance Cardiography (ICG), the VFR method uses multiple electrodes to create input for 6 independent and simultaneous voltage input channels. Our method is *not* based on impedance cardiography (ICG) algorithms, since we do not calculate any impedance measure, but use spatial patterns to retrieve volume changes of specifically the ventricles.

Some ICG-like techniques (such as e.g. Woltjer *et al*[20]) use multiple electrodes, but - in contrast to our method - still measure only one single voltage, because the leads of many of these electrodes are interconnected to form a single input channel.

Comparison of the bias and precision (2SD) of the prototype of the HCP in this study with that of other techniques as listed in e.g. [3], shows that the HCP yielded satisfactory results as a completely non-invasive technique.

### Sources of inaccuracy

There are three principal factors that may affect the validity of the relation in eq.(7):

(i) A mismatch (in the *cranial-caudal* and the *left-right* directions) between the theoretical optimal electrode positions with respect to the volunteers’ heart, corresponding to the ex-vivo calibration measurements, and the actual electrode positions that result from placing the electrodes on basis of externally visible anatomical landmarks (viz. the incisura jugularis) on a volunteer. Since considerable anatomical differences exist between the various volunteers (i.e. for each volunteer the position of the heart with respect to the incisura jugularis may be different because of a different inclination angle of the heart axis or vertical position of the heart), we have installed into our experimental HCP set-up a piece of software that prevents this mismatch by performing simulated “virtual displacements” of the heart of the ex-vivo experiments within the algorithm, in order to find an optimal match between the measured **v**_*meas*_(*τ*)and key features from the fingerprints during a brief initial calibration phase; subsequently the fingerprint vectors **f**_*A*_ and **f**_*V*_ are adapted accordingly, and the **W**_*A*_and **W**_*B*_vectors are calculated that are used from that moment onwards for that specific volunteer.

(ii) A mismatch between the distance from the skin to the heart (in the *frontal-dorsal* direction) in a human volunteer, and the distance from skin to heart in the post-mortem ex-vivo set-up. Again, considerable inter-individual anatomical variations may exist; e.g.: in the case of an obese volunteer, the thicker layer of subcutaneous fat tissue gives rise to an attenuation of the measured **v**_*meas*_(*τ*), when compared to a non-obese volunteer with the same stroke volume. This attenuation translates into a smaller *ψ*(*τ*) for the obese volunteer, which however should relate to the same ventricular volume change. Therefore, the correct ventricular volume change *V*(*τ*)−*V*(0) should be calculated as

$$V(\tau )-V(0)=K_{\mathrm{volunteer}}\psi (\tau ){\tilde{V}}_{\mathrm{calib}}$$

in which *K*_*volunteer*_ is a constant scaling factor that may be different for each volunteer. This scaling factor has been assessed by an initial calibration procedure at the beginning of the actual HCP measurements for each volunteer, using Doppler Ultrasound (see section Methods: in-vivo tests of the HCP on volunteers for details). In the future, a stand-alone initial calibration procedure to measure *K*_*volunteer*_, without the need for Doppler Ultrasound, may be incorporated into the HCP system, using an extra measurement invoking the Principle of Reciprocity: In this auto-calibration procedure, the measuring electrodes are used temporarily for current injection. In an ex-vivo study and in computer models, we have shown that, invoking the reciprocity theorem of electromagnetic fields, this procedure is capable of assessing the attenuation of signals between the heart and the skin, thus making an initial calibration using LVot Doppler unnecessary.

(iii) Errors due to the assumption of linearity in eq.(7) itself. This is a major point that deserves closer investigation. As has been indicated above, any calculation of voltage changes on basis of volume changes of blood-filled lumina such as the ventricles, is a non-linear problem, and therefore the magnitude of the inaccuracy that results from the linear assumption in eq.(7) needs to be assessed. Therefore, in the Appendix section, the (non-linear) relation between volume change and the measured data is investigated.

**Figure 9:**
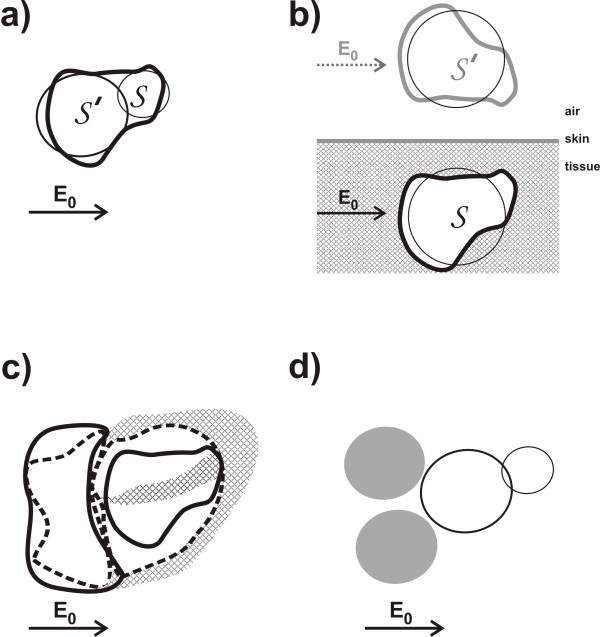
**Schematics representing various anatomical and cardiological features that contribute to the non-linearity in the relation between voltage change and volume change.*a:*** The non-spherical shape of the ventricles (feature (a) in the appendix) corresponds to an assembly of multiple spheres. In this figure, a simple representation of a ventricle, using only two spheres (indicated by *S* and *S*’), is depicted. During ventricular systole, the volume of each sphere decreases. The voltage increase, due to the volume decrease of the combination of the two spheres in close proximity, is less than the sum of the effects would have been when the effects of both spheres would have been calculated separately, without taking into account the close proximity of the two spheres. For instance, if the volume change of *S*’ increases (i.e., *S*’ becomes extra small near the end of the ventricular systole), then the strength of the incident field on *S* decreases during ventricular systole, thus diminishing the effect of the decrease of the volume of *S* on the measured voltage. Therefore, the non-linearity due to this “shadow effect” of *S*’ on *S* (and vice versa) yields a negative *η*value, viz. *η*=−0.033. ***b:*** The proximity of the heart to a boundary surface (skin-air), viz. the frontal surface of the thoracic skin. The non-linear effect due to the proximity to this boundary surface has been calculated using the Method of Image Charges [21], resulting in *η*=0.035. A decrease of *S* would entail a decrease of the mirror sphere *S*’ as well. A decrease of the volume of *S*’ would cause an increase of the strength of the incident field on *S*, thus increasing the effect that the decrease of *S* would have on the measured voltage. Hence the value of *η*is positive in this case. ***c:*** The cardiological phenomenon that a decrease of ventricular volume is generally accompanied by a simultaneous increase in atrial volume, and vice versa. In this schematic, the continuous lines represent the end-systolic situation, whereas the dotted lines refer to the end of the diastole. ***d:*** The same as in *c*, but now depicted using spheres, yielding *η*=0.107. The combined effect of the phenomena depicted in *b* and *c* yields a “worst case” of *η*=0.142. The “atrial” spheres are depicted in opaque grey.

**Figure 10:**
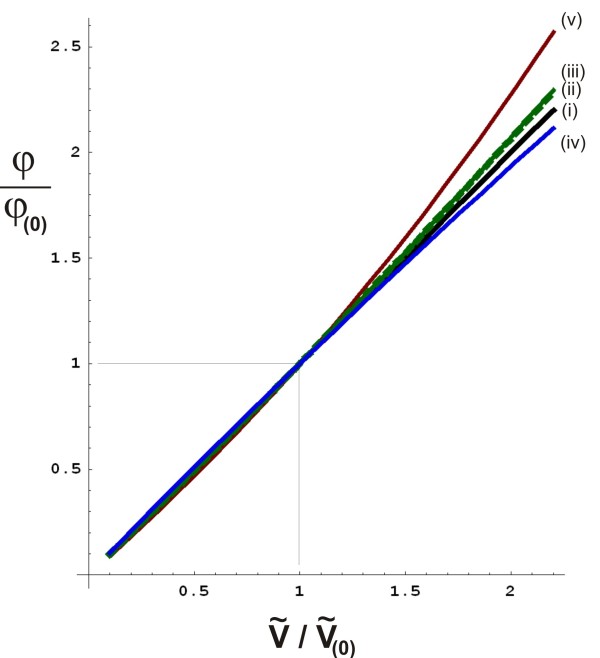
**Graphs indicating boundaries to the theoretically possible deviations from a linear relationship between relative volume change and relative voltage change (as indicated in assumption**L**,eq.(**16**)), for various basic anatomical and cardiological features as listed in the appendix.** In all graphs, the theoretical ratio *φ*/*φ*_(0)_(vertical axis, dimensionless number) has been calculated as function of the ratio $\tilde{V}/{\tilde{V}}_{\left(0\right)}$ (horizontal axis, dimensionless number), in which *φ*_(0)_is the given initial voltage change resulting from the known volume change ${\tilde{V}}_{\left(0\right)}$ as measured by the initial calibration procedure during *n*=0. In the ideal (and unrealistic) case that assumption L would hold exactly, the relation $\varphi /\varphi_{\left(0\right)}=\tilde{V}/{\tilde{V}}_{\left(0\right)}$ would hold exactly as well, as represented by the identity line (*thick continuous black line*, indicated by *(i)*). The *dotted green line*, indicated by *(ii)*, is a graph of eq. (34) with *η*=0.035, which is a second order calculation of the maximum deviation from linearity due to the presence of a boundary plane (representing the frontal surface between the conducting thorax and the insulating air) in the form of the frontal thoracic skin (feature (b) in the appendix, as depicted in Figure 9b), using the method of image charges [21]. The slender area between this dotted green line and the identity line (black solid line) represents the area containing all possible deviations from linearity (i.e., the solid black line) due to feature (b). Similarly, a graph was calculated for feature (b) using the entire perturbation series in eq.(31), resulting in the *continuous green line, (iii)*. Furthermore, the *continuous blue line (iv)* represents the maximum deviation from linearity due to the non-spherical shape of the ventricles (feature (a)), using the entire perturbation series with *η*=−0.033. Finally, the maximum deviation from linearity due to the fact that (generally) the atria are filling up when the ventricles are emptying and vice versa (feature (c)), in combination with feature (b), results in the *continuous red line (v)*, using the entire perturbation series and *η*=0.142.

In the appendix, in Figure 9, three major sources of non-linearity are depicted schematically. Using the formula (eq.(34)) derived in the appendix, confines to the maximal deviations from linearity have been calculated, yielding the graphs rendered in Figure 10.

For a volume change that equals two times the calibrated initial volume change, the non-linear effects may cause a voltage change that is maximally 14% higher than the linear approximation. Using the linear approximation therefore leads to an *overestimation* of the stroke volume of maximally 14% in this case.

For a volume change that equals half of the calibrated initial volume change, the non-linear effects may cause a voltage change that is maximally 6% lower than the linear approximation. In this case, the linear approximation therefore leads to an *underestimation* of the stroke volume of maximally 6%.

## Conclusions

A new non-invasive, operator-free, continuous ventricular stroke volume monitoring device (HCP) has been developed in our group, producing the average stroke volume (SV) for each period of 20 seconds, as well as ventricular volume-time curves for each cardiac cycle. A preliminary validation study on 7 human volunteers, using LVot Doppler as a reference technique, yielded a good correlation (Pearson correlation coefficient R=0.892) between the stroke volume according to HCP, and the stroke volume according to LVot Doppler.

The ex-vivo post-mortem measurements show that the spatial 2D distribution of voltage changes over the frontal thoracic skin due to ventricular emptying (the ventricular “fingerprint”) is clearly different from the atrial “fingerprint”.

The shapes of the ventricular volume-time curves (see Figure 4) were realistic, and consistent with the volume-time curves from the literature. Therefore, the ventricular volume-time curves have the potential of being a useful diagnostic monitoring tool as well.

## Appendix

In this appendix, the necessary theoretical underpinning of the fundamental equation 7, which constitutes the basis of the VFR method, is provided.

### A1: fundamental relation between measured voltages and volume changes

In this subsection, the validity of the assumed linearity in eq.(7) is examined. To this end, we first focuson only the ventricles and assume, for the moment, that the atria do not exist, and examine to what extent the measured changes in voltages, as combined into the vector **v**_*meas*_, may be considered to be proportional to the volume change *V*(*τ*)−*V*(0). Furthermore, we concentrate on the stroke volume of each heart beat, and enumerate the heart beats using an index (*n*) (*n*∈{1,2,3,…}), and define ${\tilde{V}}_{\left(n\right)}$ as the stroke volume of beat number *n*. Let **v**_*meas*,(*n*)_be defined as the **v**_*meas*_vector at the moment in time of maximum volume change during cardiac cycle number *n* (i.e., the end-systolic moment in cardiac cycle number *n*), and let the index (0) refer to the first heartbeat after the start of the measurements (baseline condition, *n*=0). Evidently, in the course of time after *n*=0, during later heartbeats, the stroke value may differ from the initial stroke value ${\tilde{V}}_{\left(0\right)}$

In order to evaluate the limitations to the assumed linearity in eq.(7), it is examined to what extent the following “assumption of linearity” L is valid:

(16) $$\text{assumption}\;\;L\;:\;\;\text{if}\;{\tilde{V}}_{\left(n\right)}\;=\;\lambda_{\left(n\right)}\;{\tilde{V}}_{\left(0\right)}\;\;\text{then}\;\;v_{\mathrm{meas},(n)}\;\approx \;\lambda_{\left(n\right)}\;v_{\mathrm{meas},(0)}$$

in which *λ*_(*n*)_is a scalar (representing the proportionality factor for cardiac cycle number *n*), and in which the 6 values $\varphi_{\mathrm{meas}}^{\left(k\right)}$ (*k*∈{1,2,…,6}) in **v**_*meas*_are caused by volume changes in the only volumes present, i.e. the ventricles. The assumption Lthus reads that if during cardiac cycle # (n) the stroke volume ${\tilde{V}}_{\left(n\right)}$ equals *λ*_(*n*)_ times the initial stroke volume ${\tilde{V}}_{\left(0\right)}$, then all 6 voltages in **v**_*meas*,(*n*)_would equal *λ*_(*n*)_times the initial voltages during cardiac cycle # (0) as well.

Evidently, based on the general fact that calculation of voltages from conductivity distributions is a non-linear problem, it is clear on beforehand that the assumption Lwill not be true with exact precision; hence the ≈-sign in eq.(16)

In the experiments on human volunteers, an initial calibration procedure using Doppler Ultrasound has been performed at the start of the experiment, and hence the value of the actual initial ${\tilde{V}}_{\left(0\right)}$ has been provided by Doppler Ultrasound for each volunteer.

The assumption Lcan only be valid (approximately) if, for any value of *n*>0, the shape change of the volume, during the volume change during cardiac cycle number *n*, is not completely deviant from the shape change of the volume during *n*=0. In other words, we make the assumption that sudden ruptures or other sudden abnormal patterns of volume changes are excluded. We will refer to this assumption as the “assumption of regularity R”, and will specify this assumption R more precisely later on in this subsection.

In order to assess the limits of accuracy of the assumption L, a fundamental relation between the measured relative voltage changes *φ*and volume changes of blood-filled lumina is derived below, starting with explaining the nature of the applied current field.

The oscillation frequency *f * of the measured AC voltages, which equals the oscillation frequency of the current injected into the patient via the current feeding electrodes on the legs and arms or head, is high in comparison to the physiological processes like respiration or the motion of the heart during the cardiac cycle. Furthermore, the frequency *f * is low enough to let the values of 2*Πfε* (in which *ε*is the dielectric permittivity) be negligible with respect to the differences in specific conductivity *σ* for the relevant tissues [22]. Therefore only the amplitude of the measured AC voltages needs to be considered; in our set-up each $\Phi_{\mathrm{meas}}^{\left(k\right)}(\tau )$ value was defined as the *amplitude of the oscillating voltage* measured between the electrodes *e*_*k*_ and *e*_*k*−1_ at time *τ*. This amplitude of the voltage was established every 5 milliseconds.

Directly below, a relation between the measured voltage changes *φ* and volume changes of blood-filled lumina is derived, on basis of the law of conservation of charge (the continuity equation) and Ohm’s law.

Since the current sources are applied to the head or arms and legs, there are no current injection electrodes present on the thorax. Therefore, the continuity equation for points inside the thorax reads:

(17) ∇·J(x)=0for allx∈T

in which **x** is a three-dimensional spatial coordinate ( i.e. **x** denotes a point within 3D space), and **J**(**x**) denotes the current density at point **x**, and T denotes the complete thorax, including the thoracic skin, and everything inside the thorax.

Substituting Ohms law **J**=*σ***E**in eq. (17), in which *σ*denotes the conductivity and **E** denotes the electric field, and applying the general mathematical identity

∇·(pQ)=p∇·Q+Q·∇pfor any scalarpand vectorQ

we may rewrite eq. (17) as

(18) σ∇·E+E·∇σ=0

Using $\frac{1}{\sigma }\nabla \sigma =\nabla \log\sigma$, this can be written as

(19) ∇·E=−E·∇logσ

Let the 3D vector field −∇log(*σ*(**x**))be denoted as **s**(**x**).

Therefore we now have:

(20) ∇·E(x)=E(x)·s(x)

This is an implicit equation for **E**(**x**) for a given **s**(**x**) distribution. For a model of the human anatomy in which a limited number of tissue types are defined (such as “blood”, “muscle tissue”, “bone”, etc.), the spatial conductivity distribution *σ*(**x**) is piecewise constant; i.e. the value of **s**(**x**)is zero everywhere, except at the boundary between two different tissue types. For a sharp boundary, we have [21]: $s(x)=2\hat{n}\frac{\sigma_{1}-\sigma_{2}}{\sigma_{1}+\sigma_{2}}$, in which $\hat{n}$ is the normal unit vector perpendicular to the boundary surface at point **x**, and *σ*_1_and *σ*_2_ are the specific conductivities of the two tissue types that meet at the boundary surface.

Since $\nabla \cdot E=\frac{\rho }{\varepsilon }$ (according to the Maxwell equations, in which *ρ*is the charge density and *ε*is the dielectric constant), the right-hand side of eq.(20), i.e. **E**(**x**) · **s**(**x**), may be regarded as a charge density divided by the dielectric permittivity *ε*. Therefore, points within the thorax having non-zero values of **s**(**x**)may be considered as equivalent “secondary” sources, responsible for non-zero divergence of **E** within the thorax at points on boundary surfaces. The effect of these secondary sources on the rest of the thorax (including the measuring electrodes on the thoracic skin) may be calculated using the Green function *G* of the Poisson equation.

In order to use this for the particular case of ventricular volume changes, consider eq.(20) at two instances in time:∇·**E**_0_(**x**)=**E**_0_(**x**) · **s**_0_(**x**) at time *τ*=0, i.e. at the time of the R-peak of the ECG, and ∇·**E**(**x**,*τ*)=**E**(**x**,*τ*) · **s**(**x**,*τ*)at some later instant during the same cardiac cycle (*τ*≠0). Subtracting the equation for *τ*=0from the equation for *τ*≠0, and using the extra symbols $\tilde{E}$ and $\tilde{s}$ with $\tilde{E}(x,\tau )=E(x,\tau )-E_{0}(x)$ and $\tilde{s}(x,\tau )=s(x,\tau )-s_{0}(x)$ to indicate the difference fields, yields

(21) $$\nabla \cdot \tilde{E}(x,\tau )=\underset{\left(⋆\right)}{\underset{︸}{E_{0}(x)\cdot \tilde{s}(x,\tau )}}+\underset{\left(⋆⋆\right)}{\underset{︸}{\tilde{E}(x,\tau )\cdot s(x,\tau )}}$$

This is still an implicit equation from which $\tilde{E}$ can not be calculated directly.

In eq.(21), the first term ($E_{0}(x)\;\cdot \;\tilde{s}(x,\tau )$), indicated by (⋆), represents the first order approximation, whereas $\tilde{E}(x,\tau )\cdot s(x,\tau )$, indicated by (⋆⋆), represents the self-reference in this equation, which leads to higher-order corrections in the form of a perturbation series if an exact expression for $\tilde{E}$ is derived. In the appendix, the exact solution to eq.(21) is presented in the form of such a perturbation series.

Since the purpose of this subsection is to determine to what degree of accuracy the actual volume change *V*(*τ*)−*V*(0) may be considered to be proportional to the *ψ*(*τ*)(as calculated from the measurements using eq. (11)), we first examine this proportionality on basis of the first term ($E_{0}\;\cdot \;\tilde{s}(\tau )$) alone, and, subsequently, calculate the higher-order deviations from this proportionality on basis of the perturbation series that constitutes the solution to eq.(21) as a whole.

Using the first order approximation of eq.(21), i.e. $\nabla \cdot \tilde{E}(\tau )=E_{0}\;\cdot \;\tilde{s}(\tau )$, the expression for $\tilde{E}(x,\tau )$ reads:

(22) $$\tilde{E}(x,\tau )=\nabla [G\;*\;\left(\tilde{s}(\xi ,\tau )\cdot E_{0}(\xi )\right)]$$

in which ∗ denotes the 3D spatial convolution, and ***ξ***denotes points in 3D space over which the convolution takes place, and *G* denotes the Green function of the Poisson equation ($G(x-\xi )=1/(4\Pi \sqrt{(x-\xi )\cdot (x-\xi }))$). Hence the change in the voltage difference *Φ*^(*k*)^between two electrodes *e*_*k*_and *e*_*k*−1_ (at positions **x**_*k*_ and **x**_*k*−1_ on the skin) as function of the time *τ*during one single heartbeat is calculated as:

(23) $$\Phi^{\left(k\right)}(\tau )-\Phi^{\left(k\right)}(0)=\underset{\mathrm{thorax}}{\int }\;d^{3}\xi \left[G(x_{k-1}-\xi )-G(x_{k}-\xi )\right]\tilde{s}(\xi ,\tau )\cdot E_{0}(\xi )$$

in which *∫*_*thorax*_*d*^3^*ξ*denotes integration over 3D space. with ***ξ***denoting points in 3D space. From this equation, a basic relation between voltage changes and volume changes can be derived, as is indicated in Figure 8.

Consider, as a simplified example, the boundary of a single volume of blood within an environment of tissue. This boundary is rendered in Figure 8a for the initial situation at *τ*=0. The value of **s**(**x**,0)is zero everywhere, except at the boundary. At a later instant *τ*_*ES*_during the same cardiac cycle (in which the subscript “*ES*” refers to “end-systole”), the volume of blood has reached its maximum volume change.

As is explained in the legend of Figure 8, the subtraction of Figure 8a from Figure 8b yields Figure 8c, in which the equivalent charge distributions $\tilde{s}\cdot E_{0}(x)$ are rendered that give rise to *Φ*^(*k*)^(*τ*_*ES*_)−*Φ*^(*k*)^(0) in eq.(23). All situations rendered in Figure 8 are from one single heartbeat, i.e. cardiac cycle number *n*. The relation with maximum volume change (i.e., the stroke volume ${\tilde{V}}_{\left(n\right)}$ of cardiac cycle number *n*) is now indicated in Figure 8d. The volume change ${\tilde{V}}_{\left(n\right)}=V(\tau_{\mathrm{ES}})-V_{0}$, in which *V*(*τ*_*ES*_) relates to Figure 8b, and *V*_0_relates to Figure 8a, can be calculated as the sum of the set of small grey rectangular beams in Figure 8d. Let the small grey beams be enumerated using the index *i*. Since the direction of the small grey beams, and hence of *δ***A**_*i*_, may be chosen freely when calculating a volume, we choose *δ***A**_*i*_to be parallel to ${\hat{e}}_{0}$, in which ${\hat{e}}_{0}$ is the direction parallel to **E**_0_, with

$$E_{0}=E_{0}{\hat{e}}_{0}\text{and}\delta A_{i}=A_{i}{\hat{e}}_{0}\text{and}h_{i}=h_{i}{\hat{e}}_{0}$$

Therefore the volume change now reads

(24) $${\tilde{V}}_{\left(n\right)}=V(\tau_{\mathrm{ES}})-V(0)=\underset{i}{\sum }h_{i}\cdot (\delta A_{i})=\underset{i}{\sum }h_{i}A_{i}$$

The model depicted in Figure 8 can incorporate volume changes in a direction perpendicular to ${\hat{e}}_{0}$ as well: If the volume is becoming smaller in a direction perpendicular to ${\hat{e}}_{0}$ , then some rods near the boundary become gradually shorter until they vanish completely (when the length of the rod becomes zero). If the volume is becoming larger in a direction perpendicular to ${\hat{e}}_{0}$ , some vanished rods with length zero obtain non-zero length again (effectively adding new rods at the boundary of the volume).

Similarly, the charges **s**·**E**_0_(**x**)may be considered to be a set of dipoles $p_{i}=q_{i}h_{i}{\hat{e}}_{0}$ (as explained in Figure 8), enumerated by the index *i*, with dipole length *h*_*i*_ and charge *q*_*i*_.

We will now examine the proportionality between the volume change ${\tilde{V}}_{\left(n\right)}=V(\tau_{\mathrm{ES}})-V(0)$ and the voltage differences measured on the thorax resulting from the set of dipoles $p_{i}=q_{i}h_{i}{\hat{e}}_{0}$.

At this point, we provide a more precise definition of the “assumption R” (the assumption of regularity in the volume changes) that has been mentioned at the beginning of this subsection:

(25) $$\text{assumption}R:\text{if}{\tilde{V}}_{\left(n\right)}=\lambda_{\left(n\right)}{\tilde{V}}_{\left(0\right)},\text{then}\forall i:h_{i,(n)}=\lambda_{\left(n\right)}h_{i,(0)}$$

With this assumption R, and the dipole modeling described above, the voltage change *Φ*^*k*^(*τ*_*ES*_)−*Φ*^*k*^(0)in eq.(23) for cardiac cycle *#n*may be written as:

(26) $$\Phi_{\left(n\right)}^{k}(\tau_{\mathrm{ES}})-\Phi_{\left(n\right)}^{k}(0)=\underset{i}{\sum }\left[G_{\mathrm{dipole}}(x_{k}-\xi_{i})-G_{\mathrm{dipole}}(x_{k-1}-\xi_{i})\right]\cdot {\hat{e}}_{0}h_{i,(n)}q_{i}$$

in which $G_{\mathrm{dipole}}(x)=\hat{x}/(4\Pi \varepsilon (x\cdot x))$.

Substituting *h*_*i*,(*n*)_=*λ*_(*n*)_*h*_*i*,(0)_from eq. (25) and *q*_*i*_=2*εE*_0_*A*_*i*_(*σ*_*blood*_−*σ*_*tissue*_)/(*σ*_*blood*_ + *σ*_*tissue*_)[21] in the simple example of Figure 8, this yields :

(27) $$\begin{array}{lll} \;\Phi_{\left(n\right)}^{k}\left(\tau_{\mathrm{ES}}\right)-\Phi_{\left(n\right)}^{k}(0) & =\underset{i}{\sum }\left[G_{\mathrm{dipole}}\left(x_{k}-\xi_{i}\right)-G_{\mathrm{dipole}}(x_{k-1}-\xi_{i})\right]\cdot {\hat{e}}_{0}h_{i,(n)}\; & \;\\ & \;\times \left(2A_{i}E_{0}\frac{\sigma_{\mathrm{blood}}-\sigma_{\mathrm{tissue}}}{\sigma_{\mathrm{blood}}+\sigma_{\mathrm{tissue}}}\right)=2\lambda_{\left(n\right)}E_{0}\frac{\sigma_{\mathrm{blood}}-\sigma_{\mathrm{tissue}}}{\sigma_{\mathrm{blood}}+\sigma_{\mathrm{tissue}}}\underset{i}{\sum }h_{i,(0)}A_{i}\; & \;\\ & \;\times \left[G_{\mathrm{dipole}}(x_{k}-\xi_{i})-G_{\mathrm{dipole}}(x_{k-1}-\xi_{i})\right]\cdot {\hat{e}}_{0}\; \end{array}$$

from which it becomes apparent that, having used the assumption of regularity R(eq. (25)) and the first order approximation in eq.(22), $\Phi_{\left(n\right)}^{k}(\tau_{\mathrm{ES}})-\Phi_{\left(n\right)}^{k}(0)$is indeed proportional to $\lambda_{\left(n\right)}\underset{i}{\sum }h_{i,(0)}A_{i}$, and hence assumption Lholds in this case. Furthermore, eq.(27) shows that $\Phi_{\left(n\right)}^{k}(\tau_{\mathrm{ES}})-\Phi_{\left(n\right)}^{k}(0)$is proportional to the incident field strength *E*_0_as well.

As has been indicated before however, it is clear on beforehand that the assumption L will not be true with exact precision, due to the fact that the relation between volumes and voltages in fundamentally non-linear. The strength of the non-linear effects will be examined in the next subsection (A2).

### A2: non-linear effects

In the subsection above, the first order explicit equation (22) has been derived from the general implicit equation (21), by neglecting the self-referential term indicated by (⋆⋆)in eq.(21). The exact version of equation (22) however, i.e. without neglecting the self-referential term indicated by (⋆⋆) in eq.(21), reads:

(28) $$\tilde{E}(x)=\nabla \underset{\mathrm{thorax}}{\int }\;d^{3}\mathrm{\xi G}(x-\xi )\left(E_{0}(\xi )\cdot \tilde{s}(\xi )+s(\xi )\cdot \tilde{E}(\xi )\right)$$

and hence the voltage difference between the two electrodes *e*_*k*_and *e*_*k*−1_equals:

(29) $$\;\;\;\Phi^{\left(k\right)}(\tau )-\Phi^{\left(k\right)}(0)=\underset{\mathrm{thorax}}{\int }\;d^{3}\xi \left[G(x_{k-1}-\xi )-G(x_{k}-\xi )\right]\left(E_{0}(\xi )\cdot \tilde{s}(\xi )+s(\xi )\cdot \tilde{E}(\xi )\right)$$

In order to transform equation (28) into an explicit equation for $\tilde{E}$ (and hence solving eq.(29) as well), an iterative substitution method is used that is comparable to the iterative substitution that produces the Born-Neumann series from the Lippmann-Schwinger equation, i.e.: the symbol $\tilde{E}$ appearing in the right-hand side of eq.(28) is replaced by the expression that is formed by the entire right-hand side of eq.(28). This results in a new equation in which $\tilde{E}$ appears again near the end of the right side of the resulting equation, and hence the substitution procedure can be repeated, which yields an iterative substitution procedure.

In the top line of eq.(30) the result of the iterative substitution is rendered, and subsequently “expanded” by distribution through parentheses (in the second line of eq.(30)), yielding a perturbation series:

(30) $$\begin{array}{lll} \tilde{E}\;= & \;\nabla G\;*\;\left(E_{0}\;\cdot \;\tilde{s}\;+\;s\;\cdot \;\nabla G\;*\;\left(E_{0}\;\cdot \;\tilde{s}\;+\;s\;\cdot \;\nabla G\;*\;\left(\ldots \ldots \right)\right)\right)\; & \;\\ = & \;\nabla G\;*\;\;\underset{A}{\underset{︸}{\left(\;\overset{\infty }{\underset{\vartheta =0}{\sum }}{\left[s\;\cdot \;\nabla G\;*\;\right]}^{\vartheta }\;\right)}}\;\;(E_{0}\;\cdot \;\tilde{s})\; \end{array}$$

in which, again, the symbol ∗denotes the convolution operator in three dimensions.

The voltage difference between the two electrodes *e*_*k*_and *e*_*k*−1_ hence reads:

(31) $$\;\Phi^{\left(k\right)}(\tau )-\Phi^{\left(k\right)}(0)\;=\;\underset{\mathrm{thorax}}{\int }\;d^{3}\xi \;\;\;\left[G(x_{k-1}-\xi )-G(x_{k}-\xi )\right]\;\underset{A}{\underset{︸}{\left(\;\overset{\infty }{\underset{\vartheta =0}{\sum }}{\left[s\;\cdot \;\nabla G\;*\;\right]}^{\vartheta }\;\right)}}\;\;(E_{0}\;\cdot \;\tilde{s})$$

An important parameter for the assessment of convergence of the series Ain eq. (30) and eq.(31)) is the fraction $\frac{|\tilde{E}(x)|}{|E_{0}(x)|}$.

If the fraction $\frac{|\tilde{E}(x)|}{|E_{0}(x)|}$ is small compared to one, then the higher order (large *ϑ*) terms in (31) become rapidly irrelevant with increasing *ϑ*.

Since the cardiac motion causes only a small distortion of the applied current field, we have that $\frac{|\tilde{E}(x)|}{|E_{0}(x)|}$ is indeed small compared to one, as can be shown for the theoretical case of a single spherical volume representing a ventricle in a homogeneous surroundings. For this theoretical case, the maximum value of $\frac{|\tilde{E}(x)|}{|E_{0}(x)|}$ outside the sphere can be calculated exactly, and equals 0.14 for the conductivity values of blood and muscle tissue, and is reached near the surface of the sphere on the central axis of the sphere parallel to the incident field **E**_0_. For most other positions **x**, the value of $\frac{|\tilde{E}(x)|}{|E_{0}(x)|}$ is much lower.

We now use eq.(31) to examine the non-linear character of the relation between volume changes and the measured *Φ*^(*k*)^(*τ*) − *Φ*^(*k*)^(0) by adding various anatomical and cardiological features, starting with the above mentioned simple theoretical case of a single spherical volume of blood (with *σ*_*blood*_) inside an infinite homogenous surroundings (with specific conductivity *σ*_*tissue*_). From the literature [21] it is known that, in this specific case, the electric field in the surroundings of the sphere is exactly equal to the sum of the incident field plus the field from a single dipole located at the center of the sphere.

As a result, the *Φ*^(*k*)^(*τ*) − *Φ*^(*k*)^(0)will be exactly proportional to the volume of the sphere.

Non-linear effects however do occur in A in eq.(31) if other objects are present besides the single sphere. Evidently, the true deviation from linearity in a complex realistic anatomical model can not be rendered in an explicit analytical expression. However, it is possible to derive analytically a set of boundaries that confine the possible deviations from linearity to a well-defined and narrow range for each specific anatomical and cardiological feature. In order to do so, these specific anatomical and cardiological features are translated into sets of spheres (as will be explained below). First, let the change in the incident field on a sphere *S*, caused by the presence of *another* sphere *S*’, be denoted by $\left(\overset{˘}{E}(x)\right)$. In order to calculate the confines of the range of the possible deviations from linearity, the following step is taken: for each point on the surface of *S*, the vector $\left(\overset{˘}{E}(x)\right)$ of the incident field on *S*, due to the presence of the other sphere *S*’, is replaced by the same vector $\left({\overset{˘}{E}}_{\max}\right)$, in which $\left({\overset{˘}{E}}_{\max}\right)$ is the maximum (“worst case”) value of all original $\left(\overset{˘}{E}(x)\right)$ vectors on the surface of sphere *S*. This produces a stronger non-linearity than in the case of an exact calculation, and hence provides an analytical expression for the upper limit to the strength of the non-linear effects.

The following three fundamental anatomical and cardiological features are considered that each produce a specific deviation from linearity (see Figure 9): 

 (a) The non-spherical shape of the ventricles (Figure 9a);

 (b) The proximity of the heart to a boundary surface (skin-air), viz. the frontal surface of the thoracic skin (Figure 9b);

 (c) The cardiological phenomenon that, e.g. during the rapid filling phase of the ventricles, the volume changes in the atria tend to be comparable to the volume changes in the ventricles, but have the opposite sign. In a completely linear model, neglecting all higher order terms (i.e., neglecting the term indicated by (⋆⋆) in eq.(21) ), the volume changes in the atria would contribute to *onlyα*(*τ*)in eq.(7), and the volume changes in the ventricles would contribute to *onlyψ*(*τ*). However, indirectly, a volume change in the atria will change also the way that a volume change in the ventricles is perceived by the measuring electrodes, because a volume change in the atria will change the strength of the *incident field* on the ventricles, giving rise to a second order effect. (Figure 9c)

As an example, consider the following calculation of an upper limit to the second order deviation from linearity for feature (b). In Figure 9b, the feature of the proximity of the heart to a boundary surface (skin-air), i.e. feature (b), is rendered schematically. The method of image charges [21] is used to replace the skin-air boundary by a mirror image of the heart in a homogenous continuum.

In the spherical modeling, increasing the stroke volume (as described by $\tilde{s}$ ) with a factor *λ* is equivalent to replacing $\tilde{s}$ with $\lambda \tilde{s}$ in its effect on the measuring electrodes. Furthermore, the $\left(\overset{˘}{E}(x)\right)$ near sphere *S*, caused by the presence of the mirror sphere *S*’, is proportional to the volume of the mirror sphere *S*’, and therefore also proportional to *λ*, and, as a result, the maximum $\left({\overset{˘}{E}}_{\max}\right)$ is proportional to *λ*as well. Since $\left(|\overset{˘}{E}(x)|\right)$ is also proportional to ∣**E**_0_∣, the $\left(|{\overset{˘}{E}}_{\max}|\right)$ may be written as *ληE*_0_, in which *η*depends on the distance between the spheres. As a result, on basis of eq.(29), the expression ${\left[\Phi_{\left(n\right)}^{\left(k\right)}(\tau_{\mathrm{ES}})-\Phi_{\left(n\right)}^{\left(k\right)}(0)\right]}_{\max}$, representing a “worst case” estimation for $\left(\Phi_{\left(n\right)}^{\left(k\right)}(\tau_{\mathrm{ES}})-\Phi_{\left(n\right)}^{\left(k\right)}(0)\right)$ that has a maximum deviation from linearity, yields:

(32) $$\begin{array}{lll} {\left[\Phi_{\left(n\right)}^{\left(k\right)}(\tau_{\mathrm{ES}})-\Phi_{\left(n\right)}^{\left(k\right)}(0)\right]}_{\max} & =\underset{\mathrm{thorax}}{\int }d^{3}\xi \;\;\left[G\left(x_{k-1}-\xi \right)-G\left(x_{k}-\xi \right)\right]\; & \;\\ & \;\times \left(E_{0}(\xi )\;\cdot \;(\lambda_{\left(n\right)}\tilde{s}(\xi ))+(s_{0}(\xi )+\lambda_{\left(n\right)}\tilde{s}(\xi ))\;\cdot \;{\overset{˘}{E}}_{\max}\right)\; & \;\\ & =\underset{\mathrm{thorax}}{\int }d^{3}\xi \left[G(x_{k-1}-\xi )\;-\;G(x_{k}-\xi )\right]\left(\underset{\propto \;\lambda_{\left(n\right)}E_{0}}{\underset{︸}{E_{0}(\xi )\;\cdot \;(\lambda_{\left(n\right)}\tilde{s}(\xi ))}}\right)\; & \;\\ & \;+\left(\underset{\propto \;\eta \lambda_{\left(n\right)}E_{0}}{\underset{︸}{s_{0}(\xi )\;\cdot \;{\overset{˘}{E}}_{\max}}}+\underset{\propto \;\eta \lambda_{\left(n\right)}^{2}E_{0}}{\underset{︸}{\lambda_{\left(n\right)}\tilde{s}(\xi )\;\cdot \;{\overset{˘}{E}}_{\max}}}\right)\; \end{array}$$

in which the last term, indicated by the third horizontal brace at the far right, is proportional to the *square* of *λ*_(*n*)_, because *λ*_(*n*)_ and $\left({\overset{˘}{E}}_{\max}\right)$ are both proportional to *λ*_(*n*)_. As a result, the $\eta \lambda_{\left(n\right)}^{2}E_{0}$ under the third horizontal brace at the far right represents the second-order effect, which is a deviation from the first order linear relation between $\left(\Phi_{\left(n\right)}^{\left(k\right)}(\tau_{\mathrm{ES}})-\Phi_{\left(n\right)}^{\left(k\right)}(0)\right)$ and *λ*_(*n*)_.

Since $\varphi_{\left(n\right)}^{k}(\tau_{\mathrm{ES}})=(\Phi_{\left(n\right)}^{\left(k\right)}(\tau_{\mathrm{ES}})-\Phi_{\left(n\right)}^{\left(k\right)}(0))/\Phi_{\left(n\right)}^{\left(k\right)}(0)$(by definition, see eq.(2)) and $\left(\Phi_{\left(n\right)}^{\left(k\right)}(\tau_{\mathrm{ES}})-\Phi_{\left(n\right)}^{\left(k\right)}(0))\ll \Phi_{\left(n\right)}^{\left(k\right)}(0\right)$(empirical fact, see e.g. [15] and [23]), and $\Phi_{\left(n\right)}^{\left(k\right)}(0)\propto E_{0}$, the three proportionalities indicated under the braces in eq.(32), i.e. $\propto \;\lambda_{\left(n\right)}E_{0},\propto \;\eta \lambda_{\left(n\right)}E_{0}$, and $\propto \;\eta \lambda_{\left(n\right)}^{2}E_{0}$, respectively, entail the following equation:

(33) $${\left[\varphi_{\left(n\right)}^{k}(\tau_{\mathrm{ES}})\right]}_{\max}\;=\;a\lambda_{\left(n\right)}\;+\;\mathrm{a\eta \zeta}\lambda_{\left(n\right)}\;+\;\mathrm{a\eta}\lambda_{\left(n\right)}^{2}$$

in which *a* is an unknown scalar, and $\zeta =\frac{1}{2}\sqrt{2}$. For the special case *n*=0, i.e. the start of the measurements and the very cardiac cycle that the initial calibration refers to, we have:*λ*_(0)_=1, because by definition $\lambda_{\left(n\right)}=\frac{{\tilde{V}}_{\left(n\right)}}{{\tilde{V}}_{\left(0\right)}}$ (see eq.(16)). As a result, the unknown scalar *a* cancels out if we consider the ratio $\left(\varphi_{\left(n\right)}^{k}(\tau_{\mathrm{ES}})/\varphi_{\left(0\right)}^{k}(\tau_{\mathrm{ES}})\right)$ as function of $\frac{{\tilde{V}}_{\left(n\right)}}{{\tilde{V}}_{\left(0\right)}}$:

(34) $${\left[\;\frac{\varphi_{\left(n\right)}^{k}(\tau_{\mathrm{ES}})}{\varphi_{\left(0\right)}^{k}(\tau_{\mathrm{ES}})}\;\right]}_{\max}\;=\;\frac{{\tilde{V}}_{\left(n\right)}}{{\tilde{V}}_{\left(0\right)}}\;\cdot \;\frac{1+\eta \zeta +\eta \left(\frac{{\tilde{V}}_{\left(n\right)}}{{\tilde{V}}_{\left(0\right)}}\right)}{1+\eta \zeta +\eta }$$

Using known specific conductivities of tissues from the literature, and known typical anatomical distances and sizes, the value of *η*for feature (b) has been calculated, resulting in *η*=0.035. The corresponding graph of formula (34) using *η*=0.035is the dotted green line in Figure 10. The slender area between this dotted green line and the identity line (black solid line) in Figure 10, represents the area containing all possible deviations from linearity for feature (b).

Similarly, a graph was calculated for the entire perturbation series in eq.(31), using a geometrical power series (i.e.: $\overset{\infty }{\underset{\vartheta =0}{\sum }}\;p^{\vartheta }=1/(1-p)$ for any scalar *p*) for feature (b). This resulted in the continuous green line in Figure 10.

For each of the three features (a), (b) and (c) mentioned above, the *η* values have been calculated.

The value of *η* for the feature (a) is −0.033, and the combination of features (b) and (c), excluding feature (a) to create a worst case, yields *η*=0.142. The corresponding graphs are rendered in Figure 10 as well.

For a volume change ${\tilde{V}}_{\left(n\right)}$ that equals two times the initial volume change ${\tilde{V}}_{\left(0\right)}$, the non-linear effects may cause a voltage change that is maximally 14% higher than the linear approximation. Using the linear approximation therefore leads to an *overestimation* of the stroke volume of maximally 14% in this case.

For a volume change ${\tilde{V}}_{\left(n\right)}$ that equals half of the initial volume change ${\tilde{V}}_{\left(0\right)}$, the non-linear effects may cause a voltage change that is maximally 6% lower than the linear approximation. In this case, the linear approximation therefore leads to an *underestimation* of the stroke volume of maximally 6%.

## Abbreviations

CO: Cardiac Output; G-suit: antishock trousers (containing inflatable bladders inside the trousers); HCP: Hemodynamic Cardiac Profiler (i.e., the prototype of the new system described in this paper); LVot: Left Ventricularoutflow tract; SV: Stroke Volume; VFR method: Ventricular Field Recognition method.

## Competing interests

The authors declare that they have no competing interests. HG has received some reimbursements for advice concerning the technical setup of the experiments. Furthermore, HG, AO and CH have some financial interest in an organization that potentially may profit from publication of this manuscript in the future. HG, AO, and CH have however not been involved in the interpretation of results and conclusions in this paper.

## Authors’ contributions

MK invented the Ventricular Field Recognition method, developed the mathematical formulation and derivations in the paper, and drafted the manuscript except for parts of the ex-vivo method and electronic hardware sections. HG has developed the electronics of the HCP, wrote the electronic hardware section and reviewed the entire manuscript. FR has made essential contributions to elucidating the need for separating atrial filling effects from ventricular filling effects. MR and TH have co-planned and executed the ex-vivo measurements, and contributed to the ex-vivo methods section. AO has initialized and organized the use of G-suit stroke volume manipulation in the in-vivo experiments. RR, FK, RB, PD, AO, CH and WB have contributed to the methods, planning, and execution of the in-vivo experiments. All authors have read and approved the final manuscript.

## Author details

MK, MR, PD, and TH are with the dept. of Medical Technology of the University Medical Center Utrecht (UMCU), Utrecht, the Netherlands; RR, RB, and WB are with the depts. of Cardiology, Anatomy, and Anesthesiology, respectively, of the UMCU, Utrecht, the Netherlands; HG is with Goovaerts Instruments, Kortenhoef, NL ; AO and CH are with Hemologic BV, Amersfoort NL; FK is with ESC Medical Technology, Winschoten NL; FR is with the University Hospitals Leuven, Leuven, Belgium.
